# Stabilising the Integrity of Snake Venom mRNA Stored under Tropical Field Conditions Expands Research Horizons

**DOI:** 10.1371/journal.pntd.0004615

**Published:** 2016-06-09

**Authors:** Gareth Whiteley, Rhiannon A. E. Logan, Kam-Yin D. Leung, Fiona J. Newberry, Paul D. Rowley, John P. Dunbar, Simon C. Wagstaff, Nicholas R. Casewell, Robert A. Harrison

**Affiliations:** 1 Alistair Reid Venom Research Unit, Liverpool School of Tropical Medicine, Liverpool, United Kingdom; 2 Bioinformatics Unit, Liverpool School of Tropical Medicine, Liverpool, United Kingdom; Goethe University, GERMANY

## Abstract

**Background:**

Snake venoms contain many proteinaceous toxins that can cause severe pathology and mortality in snakebite victims. Interestingly, mRNA encoding such toxins can be recovered directly from venom, although yields are low and quality is unknown. It also remains unclear whether such RNA contains information about toxin isoforms and whether it is representative of mRNA recovered from conventional sources, such as the venom gland. Answering these questions will address the feasibility of using venom-derived RNA for future research relevant to biomedical and antivenom applications.

**Methodology/Principal Findings:**

Venom was extracted from several species of snake, including both members of the Viperidae and Elapidae, and either lyophilized or immediately added to TRIzol reagent. TRIzol-treated venom was incubated at a range of temperatures (4–37°C) for a range of durations (0–48 hours), followed by subsequent RNA isolation and assessments of RNA quantity and quality. Subsequently, full-length toxin transcripts were targeted for PCR amplification and Sanger sequencing. TRIzol-treated venom yielded total RNA of greater quantity and quality than lyophilized venom, and with quality comparable to venom gland-derived RNA. Full-length sequences from multiple Viperidae and Elapidae toxin families were successfully PCR amplified from TRIzol-treated venom RNA. We demonstrated that venom can be stored in TRIzol for 48 hours at 4–19°C, and 8 hours at 37°C, at minimal cost to RNA quality, and found that venom RNA encoded multiple toxin isoforms that seemed homologous (98–99% identity) to those found in the venom gland.

**Conclusions/Significance:**

The non-invasive experimental modifications we propose will facilitate the future investigation of venom composition by using venom as an alternative source to venom gland tissue for RNA-based studies, thus obviating the undesirable need to sacrifice snakes for such research purposes. In addition, they expand research horizons to rare, endangered or protected snake species and provide more flexibility to performing fieldwork on venomous snakes in tropical conditions.

## Author Summary

Venomous snakes use venom proteins to capture their prey. When a human is bitten, these proteins cause severe pathology and often lead to death, disability or other serious complications. Understanding the composition of snake venom is essential not only for developing more effective snakebite treatments but also for acquiring a better understanding of the biology that underpins this extraordinary group of animals. Currently, to determine the genes expressing these venom proteins requires sacrificing the snake and isolation of mRNA from the venom gland. This is undesirable for ethical reasons and limits the scope of this type of research. Encouragingly, we and others have recently demonstrated that mRNA encoding venom proteins can be obtained from lyophilized snake venom. In this study we show that venom RNA in these samples is in fact considerably degraded. We report a protocol to improve the yield of undegraded RNA (immediate addition of TRIzol reagent to the venom). We also explored the limitations of this new protocol, in terms of time and temperature applicability. We then showed that considerable isoform diversity can be captured from snake venom RNA, and show that the isoforms identified are identical to those found in the venom gland of sacrificed snakes. Our findings provide protocols enabling researchers working in a range of fields to considerably expand the scope of their research because venom composition data can now be reliably acquired from venom which in turn can be obtained from live snakes without harming the animals.

## Introduction

Venomous snakes are highly proficient predators that occupy almost all natural habitats: from desert to tropical jungle and all terrestrial environments within this range, as well as marine and freshwater bodies [1,2]. The evolution of venom proteins that cause rapid cardiovascular or neurological immobilisation and death of their prey has underpinned the predatory success of these limbless animals [2]. Snakebites on humans, on the other hand, are accidents of ‘last-resort’ defence against perceived aggression but often carry extreme consequences for the victims—venomous snakes kill over 94,000 people annually [3], most residing in remote, deeply-disadvantaged rural tropical communities [4]. Defining the protein composition of snake venoms is therefore vital for the development of new snakebite therapies (antivenom) and fundamental to basic research on venom evolution and bioactivity.

Each venomous snake species/genus has evolved a distinct repertoire of venom proteins to achieve their predatory objective [5]. Thus, the venom protein composition profile varies at every taxonomic level [6,7] and even within a species as a result of a variety of factors, including geographical origin [7–9], gender [10,11], diet [12] and age [13–15]. Some, typically the most toxic, venom protein groups show marked over-representation [6] and isoform diversity. For example, three-finger neurotoxins that can account for over 70% of cobra venom proteomes [16] are encoded by a very substantial number of sequence-distinct isoforms [17,18]. Venom transcriptomes are essential pre-requisites for research studies on, for example, the evolutionary forces driving venom protein composition [6,17,19–22] and next generation therapies [23]. The transcriptomes used in all these studies were derived from mRNA isolated from venom glands dissected from snakes that had to be sacrificed for this purpose. Aside from ethical detractions, our current dependence upon venom glands for generating transcriptomes limits our research horizons to those snake species that (i) can be readily acquired and maintained in the few research establishments with the requisite herpetological facilities and expertise or (ii) can be collected in the field. It also excludes research on the numerous snake species that are protected by national and/or international legislation (e.g., the Convention on the International Trade with Endangered Species, CITES), species that are rare/difficult to find in the field, and species that do not survive long in captivity. Furthermore, current methods do not permit studies on, for example, ontogenetic changes in venom protein composition within the life-span of an individual snake.

Shaw’s group in Belfast were the first to document that mRNA encoding toxins can be recovered from the venom or skin secretions of several species [24,25], and suggested that snake venom might be an ethically-attractive alternative to venom glands for preparing transcriptomes. The presence of nucleic acids in venom has since been exploited by using PCR-amplification of DNA in venom for snake species taxonomy [26] and to monitor gene expression dynamics over the course of venom replenishment in the venom gland [27]. The objective of the work reported herein was to examine the stability of venom mRNA recovered under tropical field conditions, and to assess the extent to which venom mRNA represents the isoformic diversity reported in venom gland transcriptomes.

## Methods

### Ethics Statement

All snakes were maintained in individual cages within the temperature, humidity and light-controlled environment of the herpetarium of the Alistair Reid Venom Research Unit, Liverpool School of Tropical Medicine. The facility and its protocols for the expert husbandry of the snakes are regularly inspected and approved by the UK Home Office and the LSTM Animal Welfare and Ethical Review Board.

### Biological Material

Venom was extracted and pooled from (i) ten, wild-caught, Nigerian puff adders (*Bitis arietans*), (ii) a single, captive bred, adult specimen of the intermediate shield-nose snake (*Aspidelaps scutatus intermedius*), (iii) six Angolan coral snakes (*Aspidelaps lubricus cowlesi*), (iv) four cape coral snakes (*Aspidelaps lubricus lubricus*) and (v) a single monocled cobra (*Naja kaouthia*). The number of snakes used in this study was the maximum possible given the limited availability of these animals in our facility. We also used data from a separate study wherein venom glands were dissected from terminally-anaesthetised specimens of *B*. *arietans*, and immediately snap frozen in liquid nitrogen.

### RNA Isolation and Quality Analysis

#### Venom

To assess the potential benefit of mRNA-stabilising reagents, we added TRIzol reagent (Life Technologies) to the venoms at a ratio of 5:1 as soon after venom extraction as safety permitted. RNA was isolated from the venom using the TRIzol Plus RNA Purification System following the manufacturer’s instructions (unless stated otherwise). To assess the effect of storage time and temperature on venom RNA stability, a sample of the *B*. *arietans* venom/TRIzol mixture was aliquoted into three equal volumes. Each sample was incubated at one of three temperatures: 4°C, 19°C or 37°C and RNA isolated (as per above) after 8, 24 or 48 hours. A sample of venom was also conventionally lyophilized and stored at 4°C until required. Each aliquot equated to 500 μL of liquid venom (equivalent to 124.66 mg lyophilized venom). All other venom samples had TRIzol added at a ratio of 5:1 and were processed using the TRIzol Plus RNA Purification System. All samples were DNase treated on column during the TRIzol Plus purification system protocol (On-Column PureLink DNase; Life Technologies) and total RNA was eluted in 30 μL nuclease free water (QIAGEN).

#### Venom gland

RNA from venom glands provided both the control for the venom RNA samples and the source for the transcriptomes from which the C-type lectin transcripts were assembled. *Bitis arietans* venom gland tissue (272 mg) was immediately homogenised under liquid nitrogen using a pestle and mortar and then a TissueRuptor (QIAGEN). Samples were DNase treated (On-Column PureLink DNase; Life Technologies) and total RNA was extracted and eluted in 30 μL nuclease free water (QIAGEN) using the TRIzol Plus RNA Purification System protocol. Venom gland transcriptomes were sequenced, assembled and annotated as previously described (Sanger sequenced EST transcriptome [28]; second generation Illumina transcriptome [29]).

### Quality Analysis

The quality and quantity of RNA isolated from conventionally lyophilized (V^LYO^) or TRIzol treated (V^TRZ^) venom samples (including after variant storage temperature and times), was compared using a Bioanalyzer (Agilent, UK). The Bioanalyzer uses the entire electrophoretic trace (electropherogram) of the RNA sample including the presence/absence of degradation products in a sample concentration-independent manner. We added 1 μL of each total RNA sample into Agilent RNA 6000 Nano/Pico Chips (following the manufacturer’s instructions) and then inserted them into the Agilent 5000 Bioanalyzer for analysis. The quality of the two ribosomal RNA peaks are used as a reliable indicator of the integrity of the other RNA molecules. Calculations are based upon quantitating the amount of fluorescent dye intercalated within the RNA, thereby efficiently discounting contaminants such as phenol (a common carry over from RNA isolation preparations) that can vitiate the accuracy of standard UV-spectrometry. Agilent’s RNA 2100 Expert software was used to calculate total RNA concentration and assign a RNA integrity number (RIN), which range from 1–10; with 10 being completely undegraded, and 1 being completely degraded RNA [30].

### Complementary DNA (cDNA) Synthesis

We isolated mRNA from 2 μL of each total RNA sample using oligo(dT) primers and processed it into single-stranded cDNA using the Superscript III first-strand synthesis system (Invitrogen). Each single-stranded cDNA sample was treated with RNase H to eliminate contaminating RNA. This process provided a stable and reproducible copy of each venom/venom gland RNA preparation.

### PCR Amplification and Sequencing

#### *Bitis arietans* primer design

Primers complimentary to genes encoding *B*. *arietans* serine proteases (SP), snake venom metalloproteinases (SVMPs), L-amino acid oxidases (LAO), C-type lectins (CTL), vascular endothelial growth factors (VEGF) and aminopeptidases (AMP) were designed (Table 1) and synthesized commercially (Sigma Aldrich). These gene families were selected as they collectively represent major and minor venom protein components, as well as long and short transcripts, and thus served to explore the limits of our experimental approach. The primer design was based upon our *B*. *arietans* transcriptome data [29].

**Table 1:** *Bitis arietans* primers.

Venom gene	Forward primer	Reverse primer	Length of PCR product (base pairs)
AMP	TTGTGGAGTGGTAATTGCG	AGCCATTCTATGTTGCTCTT	2741
SVMP	CTCCAAAATGATCCAAGTTCTC	ATTTGGAAAAGGAAGCATGG	1880
LAO	ATTCCCATCCACAATCTTC	CGACATGTTTTGGCTGATATAC	1693
VEGF	GAGAGTAGACCGCAGGGGAACG	CAAAATGGCAAACAGGGAGATGAA	934
SP	GGATCCATGGTGCTGATCAGAGTG	CTCGAGTCACCGGGGGCAAGTCGC	782
CTL	CTGCCGGGAAGGAAGGAAGACCAT	GAGCGAAGGGGGCAGAGCAGAGAT	589

#### Elapid three-finger toxin primer design

To assess whether mRNA recovered from venom includes representation of transcripts encoding multiple diverse isoforms, we generated primers complementary to the three-finger neurotoxin (3FTX) (Table 2) gene family which is abundantly expressed in the venom glands of elapid snakes (e.g., [16,17]), as well as group 1 phospholipase A_2_ (PLA_2_), CTLs, SVMPs and Kunitz-type serine protease inhibitors (KSPI) (S1 Table). However, at the outset of this study we were unsure of the gene isoform diversity of *Aspidelaps* spp. (the objective of the experiment). We therefore decided to pool the venom RNA of *N*. *kaouthia* (that has known venom gland gene isoform diversity) with that from *A*. *s*. *intermedius*, *A*. *l*. *cowlesi*, and *A*. *l*. *lubricus*, thereby ensuring the presence of multiple gene targets for PCR amplification. Furthermore, since there are no published genes encoding *Aspidelaps* 3FTXs, PLA_2_s, CTLs, SVMPs or KSPIs, our primers were designed upon conserved sequences flanking the open reading frames of published sequences from several species of the genus *Naja* (cobras), *Walterinnesia aegyptia* (desert black cobra) and *Ophiophagus hannah* (king cobra). These snakes are the closest phylogenetic relatives of the genus *Aspidelaps* [31]. To maximise the likelihood of PCR success, each forward primer was used in combination with each reverse primer to identify the best combination.

**Table 2:** Elapid three-finger toxin PCR primers.

List of forward primers	List of reverse primers
TCCAGAAAAGATCGCAAGATGA	CTAATTGTTGCATTTTTCTG
TCCGAAAAAGATCGCAAGATGA	CTATAGGTTGCATTGGTCTGTT
TCCGTTGCTGTCGGCAAGATGA	CTCAAGGAATTTAGCCACTCGT
ATGAAAACTCTGCTGCTGACCT	AAACTCAAGGAATTTAGCCACT
	GAATTTAGACATTATCAGTTG
	CTAATTGTTGCATCTGTCTGTT
	TCAGTTGCATCTGTCTGTATTG

#### PCR conditions

One μL of venom cDNA was added to 5 μL 10 x PCR buffer, 5 μL 2 mM dNTP mixture (Fisher Scientific), 2.5 μL 10 μM forward primer, 2.5 μL 10 μM reverse primer, 1 μL DreamTaq polymerase (Life Technologies) and 33 μL nuclease-free H_2_O (QIAGEN) to a final volume of 50 μL. PCR experiments were performed using a Techne TC-512 thermal cycler. Touchdown PCR conditions were as follows: Initial denaturation at 94°C for 3 min. Denaturing at 94°C, 30 sec; annealing temperature gradient 66–50°C, 1 min (-1.6°C/cycle); extension at 72°C, 1 min; 10 cycles. Denaturing at 94°C, 30 sec; annealing at 50°C, 1 min; extension at 72°C, 1 min; 25 cycles. Final extension at 72°C for 10 min. Amplicons were visualised on a 1% agarose in TBE gel, and analysed using the Bio-Rad Gel Doc System and Image Lab Software (Bio-Rad).

#### PCR amplicon sequencing

*Bitis arietans* amplicons, generated from highly stringent, gene-specific primers for SVMP, LAO, SP, VEGF and AMP, were submitted for Sanger sequencing (Beckman Coulter Inc., UK). The amplicons from the pooled elapid venoms were generated with less species- and gene-specific primers and therefore were likely to contain divergent sequences. Since we sought to capture this diversity, we purified amplicons from each PCR reaction from the agarose gel using the Promega Wizard Kit (Promega), cloned these into pCR2.1-TOPO vectors (Life Technologies), transformed chemically competent *E*. *coli* (One Shot, Invitrogen, UK) to amplify the recombinant plasmids, and submitted the purified plasmids (Miniprep Spin Kit, QIAGEN, UK) for sequencing as above. This was also completed for the *B*. *arietans* venom CTL amplicons.

### Bioinformatics

Sequencing files were trimmed of vector sequence and base calls with <30 PHRED scores using SeqTrace [32], translated using MEGA6 version 6.06 [33] and aligned using the Muscle alignment software and default settings [34]. Alignment figures were generated using BOXSHADE v3.21 using default settings.

## Results

### TRIzol Prevents Rapid Degradation of RNA in *Bitis arietans* Venom Samples

**Fig 1:**
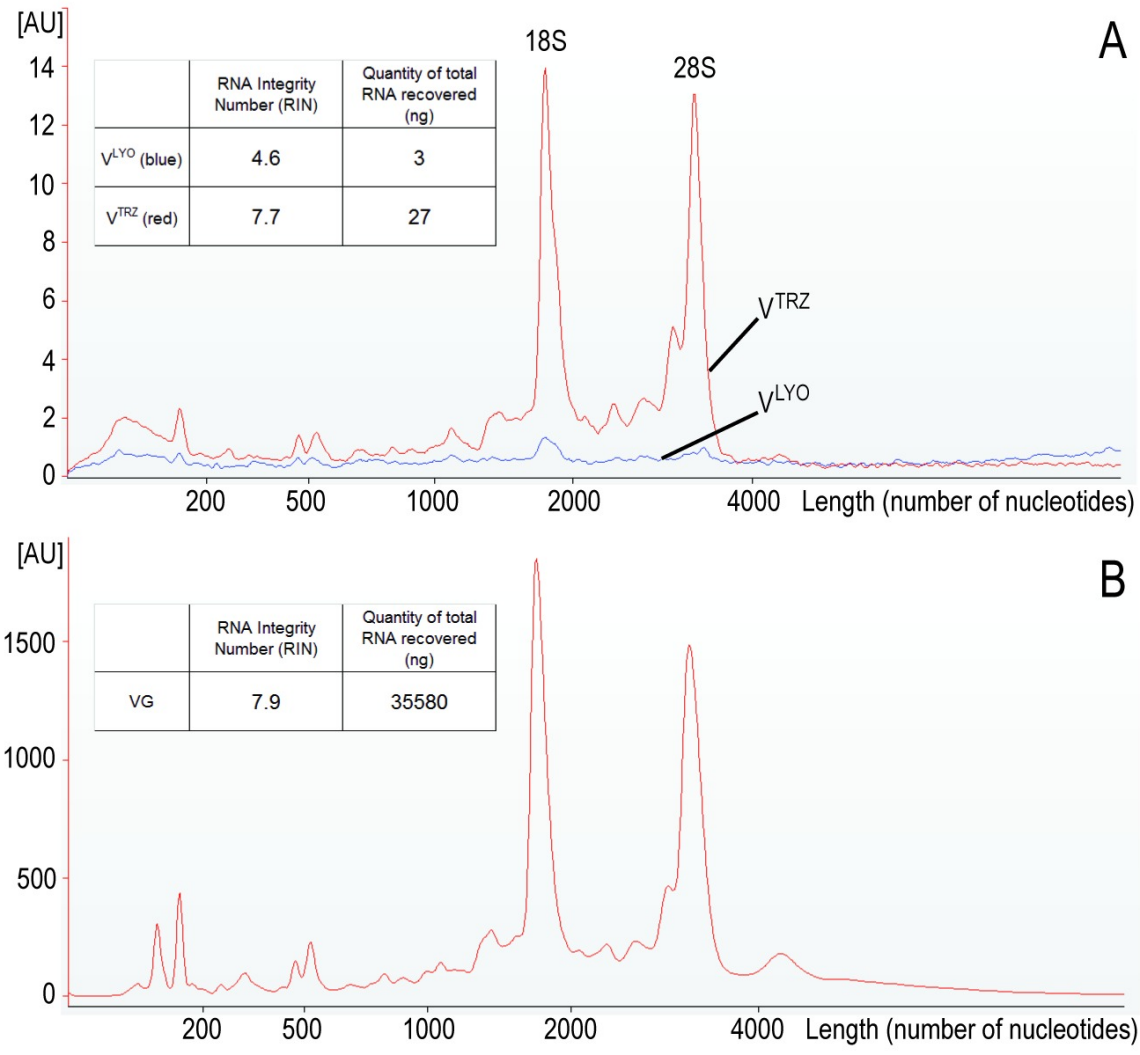
Quantitative and qualitative analysis of *Bitis arietans* total RNA isolated from lyophilized venom, TRIzol-treated venom and venom gland. (A) Lyophilized (V^LYO^, blue line), TRIzol-treated (V^TRZ^, red line) venom and (B) venom gland tissue. The y-axis is an arbitrary unit of fluorescence, and the x-axis shows the nucleotide length of the RNA. Note that the venom (A) and venom gland (B) electropherograms are presented with considerably different y-axis scale values reflecting the amount of RNA in the samples. RNA Integrity Number (RIN) and yield are shown as inserts. Key: venom gland (VG), venom (V), lyophilized (LYO), TRIzol-treated (TRZ).

We first tested the quality of RNA recovered from lyophilized venom samples (V^LYO^) because most venom collections, including the samples used in all previous publications on venom mRNA, are/will be prepared in this manner (extracted venom is frozen for 3+ hours and then lyophilized into a dehydrated powder). The RIN number of V^LYO^ was 4.6 (Fig 1A), indicating that considerable, but not complete, degradation had occurred. This was unexpected because the recovery of mRNA from venom in earlier PCR-based reports including our own [24,27], had suggested that venom mRNA must be unusually stable since mRNA is a notoriously fragile biomolecule.

As a consequence of this new observation, we next examined the integrity of RNA from venom samples to which we had added the RNA-stabilising agent TRIzol within seconds of the extraction. These TRIzol-treated venom samples (V^TRZ^) had an RIN number of 7.7 (Fig 1A), indicating that TRIzol treatment had substantially reduced RNA degradation. As a means of comparison, total RNA isolated from the venom gland of *B*. *arietans* had an RIN number of 7.9 (Fig 1B), indicating that the total RNA obtained from the V^TRZ^ was, in terms of total RNA integrity, comparable with that isolated from snap-frozen venom gland tissue.

The electropherograms of the total RNA isolated from V^TRZ^ and the venom gland (Fig 1A and 1B; red lines) show nearly identical and undegraded 18S and 28S ribosomal RNA peak profiles. In comparison, the ribosomal peak profiles for the V^LYO^ total RNA sample (Fig 1A; blue line) were only marginally above baseline. The disparity between the ribosomal peak profiles from the V^TRZ^ and V^LYO^ samples demonstrates that substantial ribosomal RNA degradation has occurred in the V^LYO^ RNA sample, and that this observation is not a function of the low amount of RNA in the V^LYO^ RNA sample.

### Addition of TRIzol Improves the Quantity of RNA That Can Be Recovered from Venom

Consistent with the RNA quality analysis, the total yield of RNA (Fig 1) obtained from V^TRZ^ (27 ng) was substantially greater than that from the same amount of V^LYO^ sample (5 ng). Nevertheless, these quantities of venom RNA are 1000-fold less than that obtained from venom gland tissue (35 μg from 272 mg of tissue). Current Next Generation Sequencing (NGS) technologies routinely require greater than 1 μg total RNA as a starting point. Thus, although the total RNA obtained from V^TRZ^ is of sufficient quality, there is insufficient quantity of total RNA for standard (non-PCR amplified) transcriptome NGS.

### Greater Abundance of Full-Length Transcripts Recovered from the Lyophilized and TRIzol-Treated Venom RNA Samples

The Bioanalyzer results suggest that total RNA is largely undegraded in V^TRZ^, but they provide no indication as to the quality/protein-encoding validity of the underlying mRNA. To investigate this, we designed primers to PCR-amplify full-length transcripts encoding *B*. *arietans* venom proteins from the V^LYO^ and V^TRZ^ samples. We selected toxin sequence templates that we had verified as differing with respect to transcript size and proportional representation [29], i.e. SVMP, LAO, SP, VEGF and AMP.

**Fig 2:**
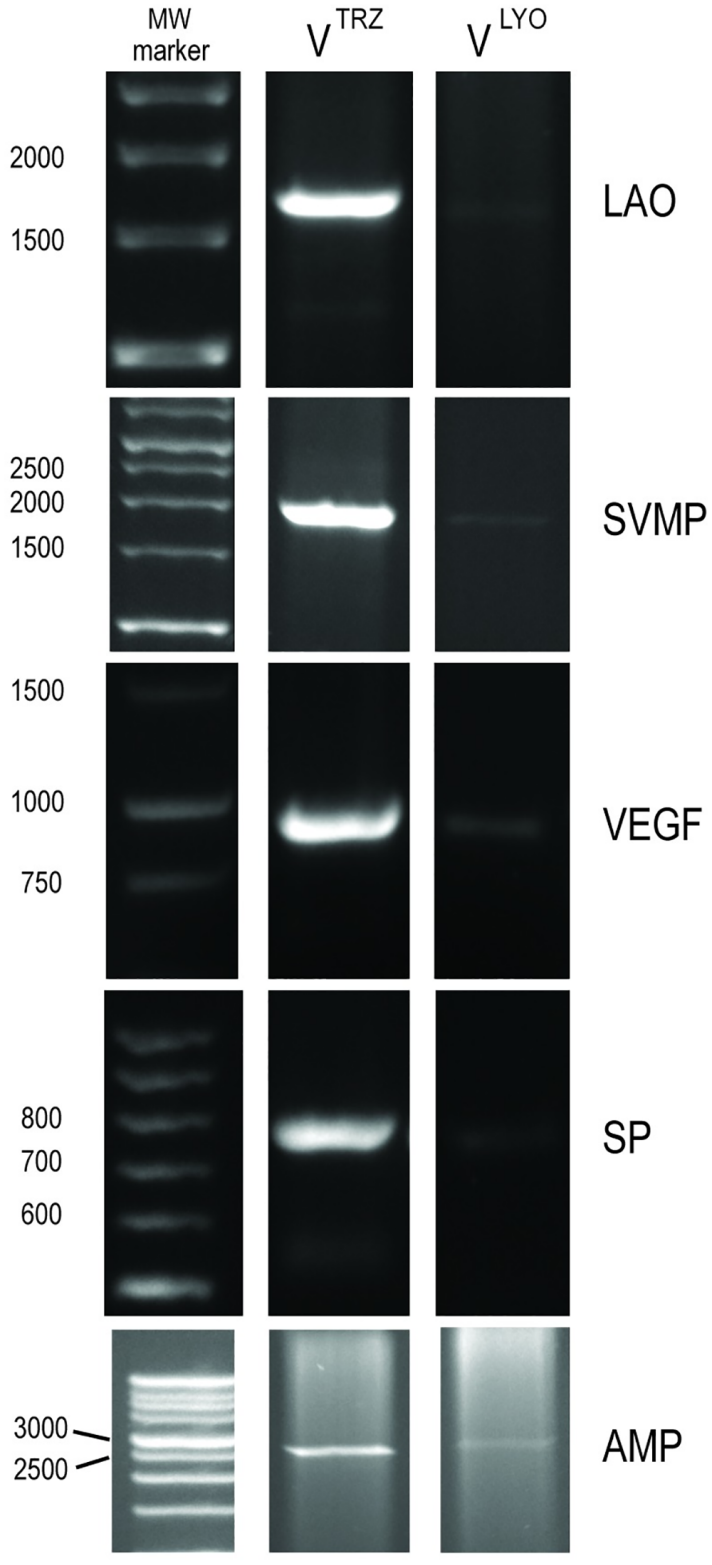
PCR amplification of *Bitis arietans* genes of distinct size and proportional representation from V^LYO^ and V^TRZ^ cDNA. Key: L-amino acid oxidase (LAO), snake venom metalloproteinase (SVMP), vascular endothelial growth factor (VEGF), serine protease (SP) or aminopeptidase (AMP). Molecular weight markers are shown on the left (base pairs).

This PCR experiment amplified intact full-length transcripts for each toxin family from both *B*. *arietans* V^LYO^ and V^TRZ^ cDNA (Fig 2). Importantly however, the intensity of amplicon bands for V^LYO^ was markedly reduced in comparison to that of the V^TRZ^ cDNA. This result reinforces the Bioanalyzer results indicating that the underlying mRNA in V^TRZ^ is less degraded than that isolated from V^LYO^.

### The Effects of Time and Temperature Storage Conditions on the Acquisition of Full-Length Transcripts from the Lyophilized and TRIzol-Treated Venom RNA Samples

Having shown the benefit of TRIzol-treating venom to acquire undegraded full-length toxin transcripts, we next explored the effects of storing V^TRZ^ samples at different temperatures (19 or 37°C) and for different durations (8, 24 and 48 hours) than those used above (4°C and 0 hours) to determine if the TRIzol-treatment protocol was suited to recovering RNA from venoms collected under field conditions.

#### Total RNA integrity analysis

**Fig 3:**
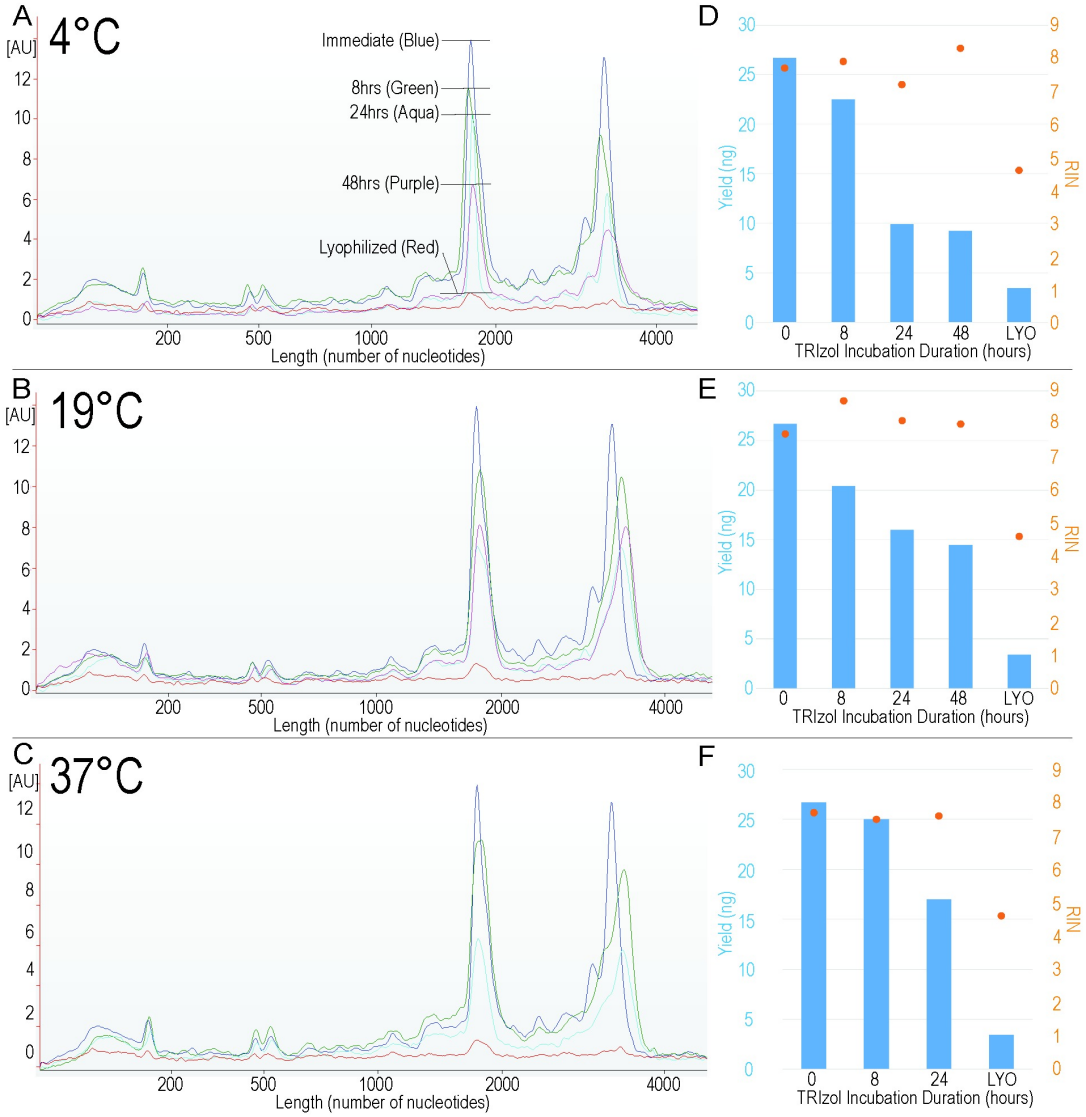
Quantitative and qualitative analysis of *Bitis arietans* total RNA isolated from V^TRZ^ for a range of TRIzol storage times and temperatures. Electropherograms for total RNA isolated from the venom of *B*. *arietans* after incubation in TRIzol at either 4°C (A), 19°C (B) or 37°C (C) for 0 hours (dark blue), 8 hours (green), 24 hours (aqua), 48 hours (purple) or after lyophilization (red). The y-axis is an arbitrary unit of fluorescence, and the x-axis shows the nucleotide length of the RNA. Total RNA yield (blue columns) and RNA Integrity Number (orange dots) after incubation at 4°C (D), 19°C (E) or 37°C (F).

Bioanalyzer electropherograms for total RNA isolated from V^TRZ^ after storage at all time and temperature conditions tested show distinct ribosomal peaks (Fig 3A, 3B and 3C) and in all cases these peaks were more defined than in those observed for V^LYO^ (red) samples. The quantity of total RNA recovered from V^TRZ^ samples decreased with extended storage periods and for all storage temperatures (Fig 3D, 3E and 3F, left axis). Again, in all cases the RNA yield from V^TRZ^ was considerably greater than that obtained from V^LYO^. Interestingly, the quality of the total RNA, as indicated by the RIN number (Fig 3D, 3E and 3F, right axis), did not change substantially for any time point or incubation temperature, and in all cases the quality of the total RNA recovered was substantially higher than that from V^LYO^.

#### mRNA integrity analysis

**Fig 4:**
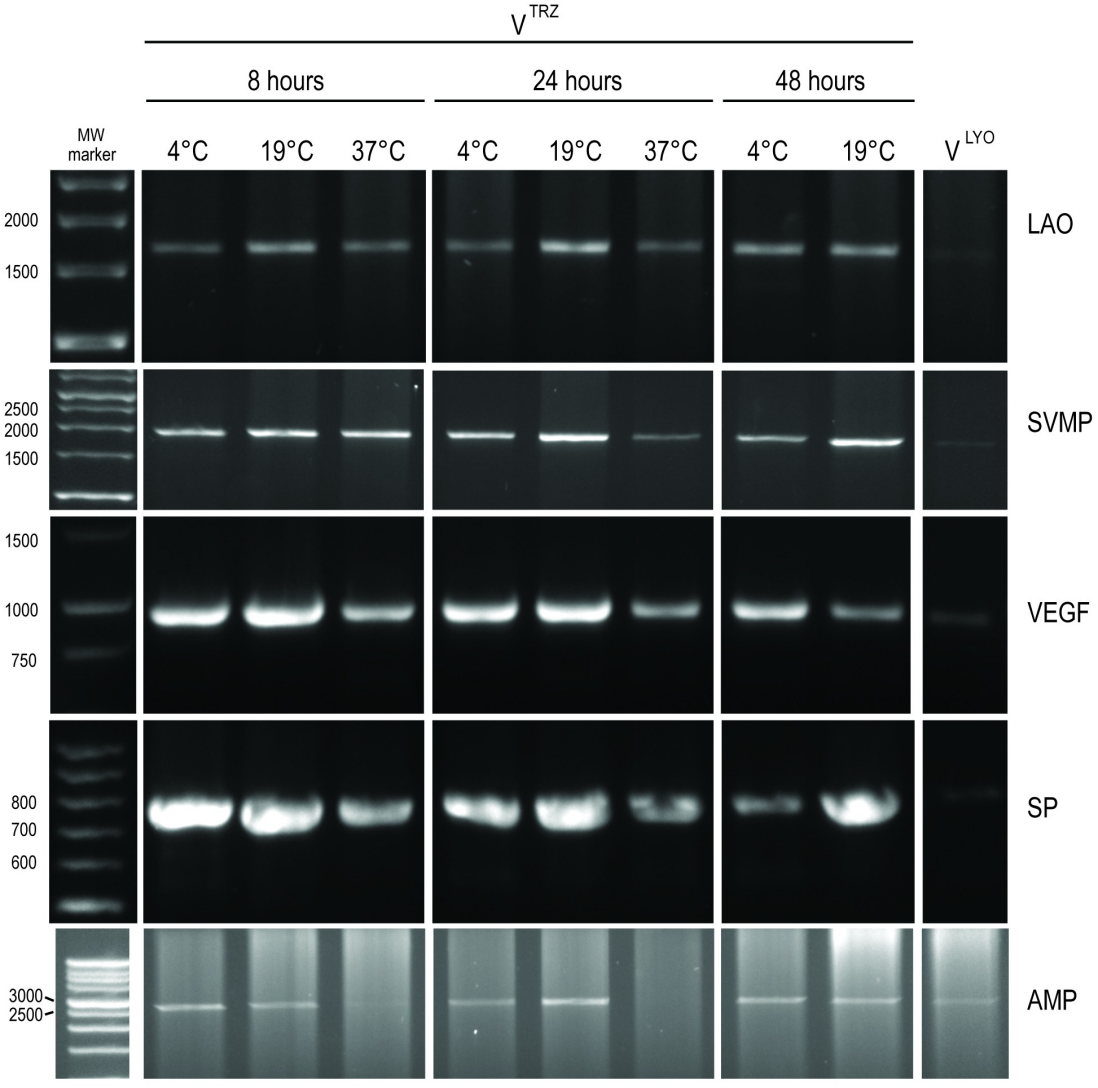
PCR amplification of *Bitis arietans* genes from V^TRZ^ after incubation with TRIzol for a range of storage times and temperatures and V^LYO^. L-amino acid oxidase (LAO), snake venom metalloproteinase (SVMP), vascular endothelial growth factor (VEGF), serine protease (SP) or aminopeptidase (AMP). Molecular weight markers are shown on the left (base pairs).

We used the same PCR-amplification protocol as above to assess the quality of mRNA in these variant time and temperature-storage venom samples (Fig 4). Treatment of venom with TRIzol preserved the quality of mRNA in venom, irrespective of duration and temperature of storage: Each amplicon encoded full-length transcripts, and with an abundance that far exceeded that of the V^LYO^ sample. Slight variability in amplicon intensity was detectable with 37°C storage showing reduced intensity relative to other temperatures. The exception to this rule was AMP, where the amplicon observed after 8 hours at 37°C was barely visible, yet an amplicon was observed in V^LYO^, suggesting that an increased temperature had resulted in the degradation of this specific transcript.

### Acquisition of Multiple, Diverse Transcripts from the TRIzol-Treated Venom RNA Samples

The most pathogenic Viperidae and Elapidae venom protein families typically exhibit the greatest gene isoform diversity and numerical representation in venom gland transcriptomes [17,28,35]. Accurately acquiring this isoform diversity poses perhaps the greatest challenge to using venom RNA as an alternative resource to venom glands for generating accurate, comprehensive transcriptomes.

To test whether this isoform diversity could be recovered from venom RNA, and to attempt the recovery of genes encoding the typically low molecular weight elapid venom proteins from venom mRNA, we prepared V^TRZ^ samples from the pooled elapid venom. Here, we PCR-amplified genes encoding the family of three-finger toxins because this gene family exhibits very considerable isoform diversity in the venom of many elapid snake species [17,18,36]. The elapid venom was treated as above for *B*. *arietans*, and the resultant cDNA subjected to PCR-amplification with a matrix of primers (Table 2) representing the range of sequences flanking the open reading frame of the 3FTX family in other elapid species. The PCR amplicons were sub-cloned into *E*. *coli* and purified plasmids sequenced.

**Fig 5:**
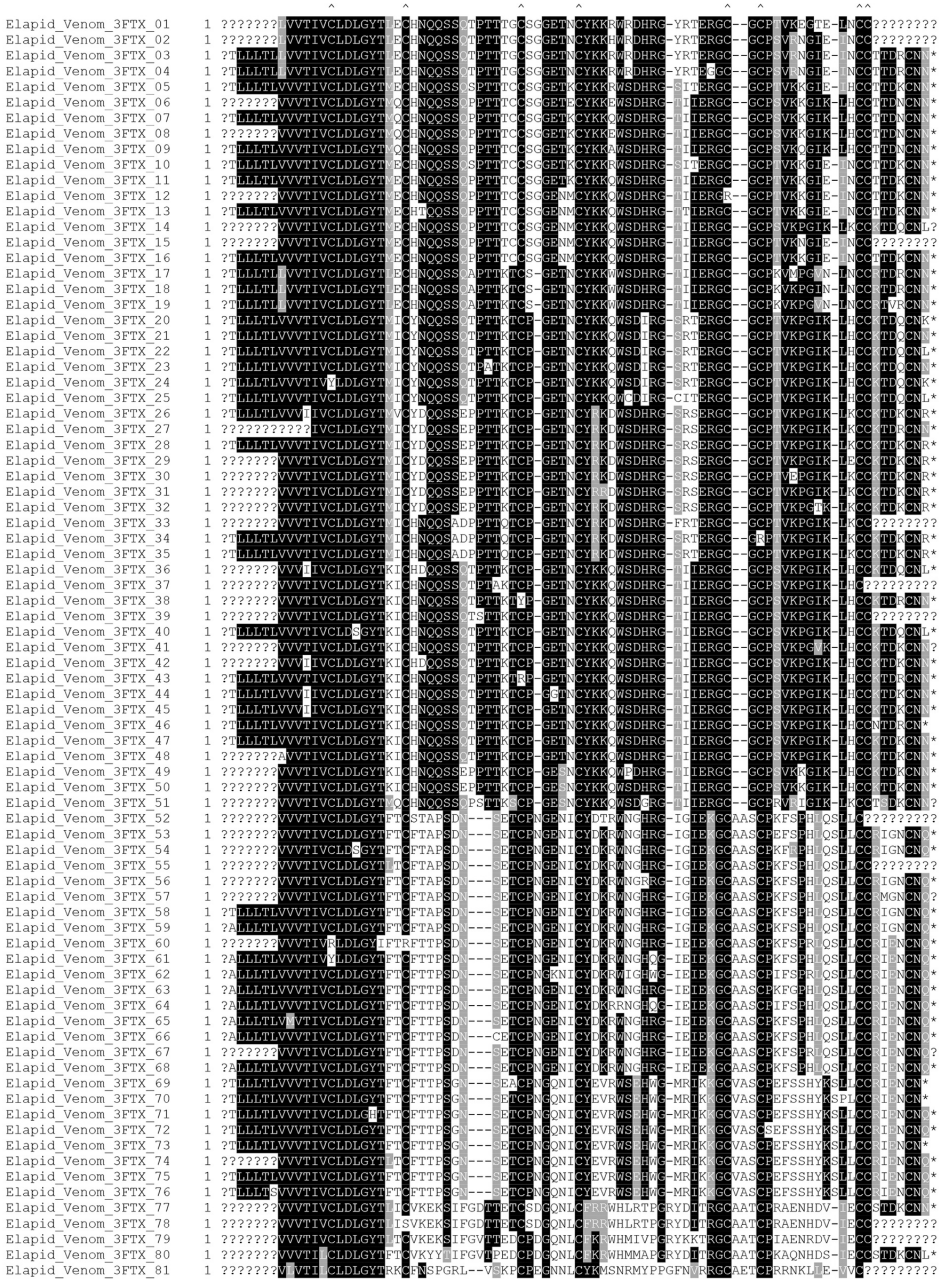
Multiple sequence alignment of elapid three-finger toxin amino acid sequences translated from transcripts from V^TRZ^. Three-finger toxin amino acid sequences translated from transcripts isolated from the V^TRZ^ of the elapid species *A*. *s*. *intermedius*, *A*. *l*. *cowlesi*, *A*. *l*. *lubricus* and *N*. *kaouthia*. Cysteine residues are highlighted with carets. Asterisks denote stop codon positions. Question marks denote unknown sequences. Black shading indicates identical amino acids, grey shading indicates similar amino acids and white shading indicates unrelated amino acids.

Using this approach we identified 81 unique 3FTX transcripts from the elapid venom (Fig 5). Consistent with other highly diverse snake venom gene families, the alignment of these new translated 3FTX transcripts reveals a highly (but not completely) conserved cysteine scaffold (consistent with previously described cysteine patterns), variable non-structural regions, and includes putative neurotoxins, muscarinic toxins and cytotoxins [18]. Many other isoforms were also identified for the PLA_2_, CTL, SVMP and KSPI venom protein families (S1 Fig).

### Sequence Comparison of C-Type Lectin Sequences Obtained Using a Variety of Techniques from Varied Sources

**Fig 6:**
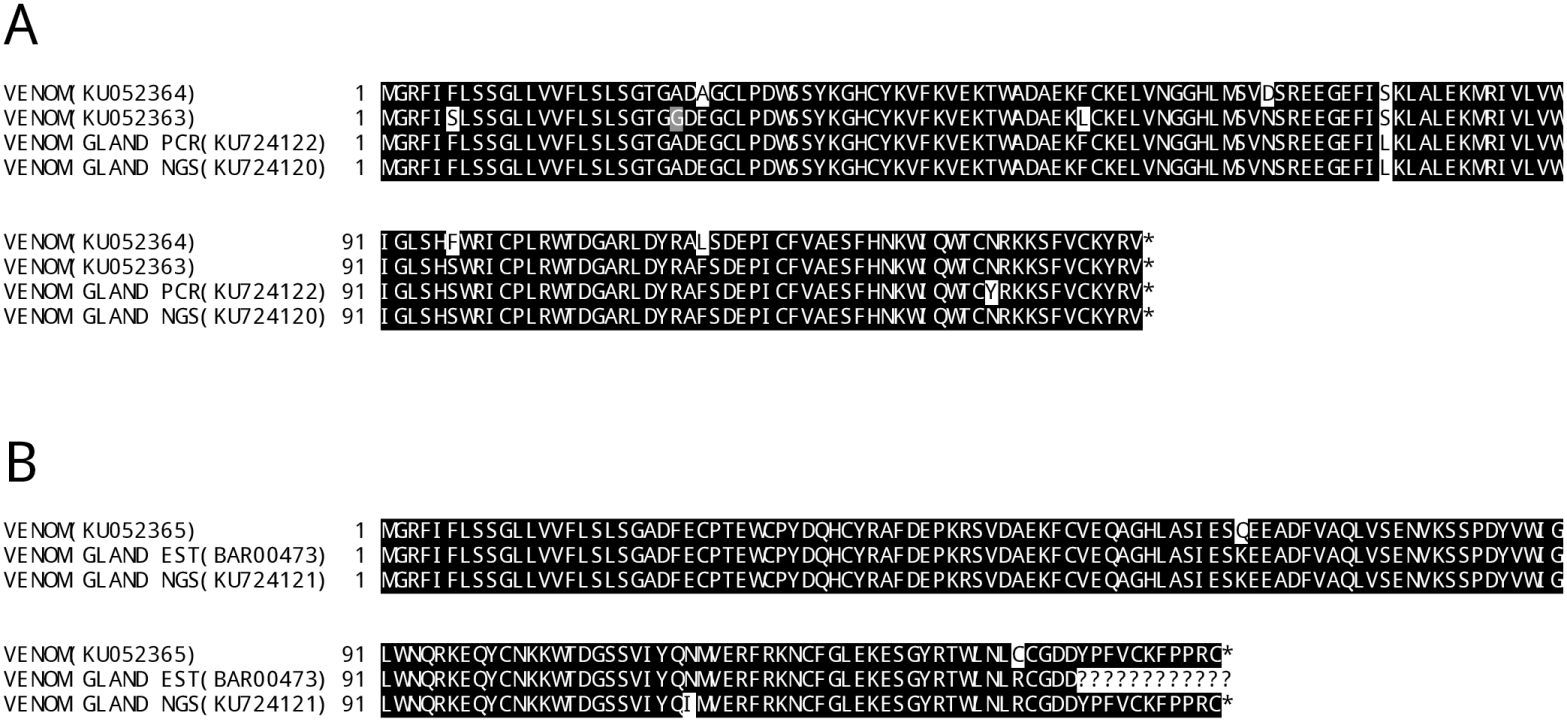
Multiple sequence alignment of C-type lectin amino acid sequences from transcripts isolated from V^TRZ^ and venom gland (PCR/EST/NGS). Two separate alignments (A, B) of C-type lectin isoforms obtained from *B*. *arietans* venom (GenBank accession numbers KU052363-KU052365) with those found via PCR from i) the venom gland (KU724122), ii) EST sequences ([6]), and iii) contigs assembled from Illumina MiSeq reads, prepared and assembled as previously described [29] (KU724120, KU724121). Key: Expressed Sequencing Tags (EST); Next generation sequencing (NGS). Asterisks denote stop codon positions. Question marks denote unknown sequences. Black shading indicates identical amino acids, grey shading indicates similar amino acids and white shading indicates unrelated amino acids.

To demonstrate that the V^TRZ^ RNA comprises the same range of isoforms obtained from (i) different sequencing techniques, (ii) from venom glands and (iii) from Viperidae venoms, we compared PCR-derived *B*. *arietans* CTL genes obtained from V^TRZ^ RNA to those obtained from venom gland samples (a) after PCR amplification, (b) as Expressed Sequence Tags (EST) from a cDNA library, and (c) as NGS sequences. As anticipated, the V^TRZ^ CTL sequences included analogues (98–99% nucleotide sequence identity) of the isoforms recovered from venom gland and by whatever sequence protocol used (Fig 6). The slight sequence differences observed are likely a result of inter-individual sequence differences, as the venom was obtained from a different set of *B*. *arietans* individuals than those used for the venom glands.

## Discussion

Messenger RNA has been shown, using PCR-amplification techniques, to be present in the venom or skin secretions of several venomous animals [24,25,27]. Whilst very encouraging with respect to using venom for transcriptomic studies, we were concerned that the transcript-amplification prowess of PCR may have overstated the ability of mRNA to remain stable in venom, which contains several nucleases. In this study, we therefore decided to determine the quantity and protein-encoding integrity of venom mRNA.

We first demonstrated that RNA recovered from lyophilized venom (V^LYO^) was in fact extensively degraded. We therefore modified the experimental approach, by omitting the lyophilization step and instead treating venom with TRIzol immediately after extraction (V^TRZ^). We show here that the complete coding regions of the transcripts can be PCR amplified from both V^LYO^ and V^TRZ^ samples (Fig 2), but with considerably more success from the V^TRZ^ samples. This represents the first case of the PCR amplification of the entirety of the coding sequence for several transcripts from snake venom. In combination, the Bioanalyzer and PCR results suggest that Bioanalyzer electropherograms alone can, with some confidence, be used to discern the protein-encoding integrity of venom mRNA.

We have also shown that venom can be stored in TRIzol for up to 48 hours at 4–19°C (and for 8 hours at 37°C) at minimal cost to RNA degradation (Figs 3 and 4). We also demonstrated that the RNA recovered using this approach is of sufficient quality to recover and characterise multiple toxin isoforms using a PCR-based sequencing strategy (Fig 5). Finally, we also showed that, at least in the case of the C-type lectins, the isoforms identified in venom represent those derived from venom gland RNA (Fig 6). Taken together, our experimental approach facilitates the investigation of toxin-encoding genes directly from secreted venom and thus allows for the rapid acquisition of venom toxin sequence data for scientific and potential therapeutic purposes.

That RNA quality remains high, irrespective of an increase in temperature or time, likely explains why the majority of transcripts were successfully PCR amplified. These results suggest that the incubation of venom in TRIzol helps to reduce degradation of RNA, including mRNA, but that it comes at a duration-to-yield cost. For example, incubation of venom in TRIzol at 19°C for all durations resulted in a greater yield of high quality RNA than was possible from V^LYO^, but as the incubation time increased the yield of total RNA decreased, although the quality remained stable. This is important, particularly for the collection of venom during fieldwork, where access to adequate storage conditions or laboratory access for sample processing are often very limited. Our study has identified the value of TRIzol processing of snake venom. This therefore suggests that other similar products may also have comparable scientific benefits, and will require the similar protocol-standardising analysis as performed here with TRIzol.

Considering the fact that snake venom contains a diverse array of destructive nucleases and phosphodiesterases [37] and is an acidic environment [14,38], the apparent presence of mRNA had originally been thought to be paradoxical [27]. Given that this environment is likely destructive to RNA, it had been suggested that venom mRNA may have unique properties that prevented it from degradation. However, the increased quality and quantity of RNA recovered from V^TRZ^ over V^LYO^ (Figs 1 and 2) suggests otherwise, indicating that the RNA recovered from lyophilized venom is in fact extensively degraded. It is likely that any previously observed success with PCR using V^LYO^ is the result of the ability of PCR to amplify trace amounts of residual RNA that have escaped degradation, or the amplification of fragments of specific transcripts (and not the entire transcripts).

Here, we cloned and sequenced full-length venom transcripts from pooled elapid snake venom, demonstrating that considerable isoform variability can be detected using our experimental approach. Eighty-one novel 3FTX sequences were obtained (Fig 5). In addition, the results also provided evidence that elapid venom contains RNA, as well as the first case of any 3FTX transcript being identified in snake venom. The successful amplification of equivalent C-type lectin isoforms from both *B*. *arietans* venom and venom gland (Fig 6) suggests that the isoforms detected from venom represent those found in the venom gland, regardless of how they were acquired (conventional PCR, EST or NGS). In combination these observations suggest that snake venom is a viable alternative to the venom gland as a source of both isoformic diversity and venom-gland representative data.

Consequentially, toxin transcript expression can be monitored over time to detect changes as a result of ontogenetic stages of development, diet or adaptation to captivity—issues that likely effect antivenom efficacy—and which could not possibly be monitored if an animal had to be sacrificed to obtain its RNA.

Our results provide a novel extension of the utility of venom derived mRNA, but working with such samples is not without its limitations. Currently, the amount of RNA recovered from the venom of a single snake is very low, and is far less than that required for current standard NGS approaches (e.g. transcriptomics). The pooling of venom sourced from many snakes of the same species would reduce this limitation, but would of course impart its own set of limitations on subsequent analysis, particularly given reports of intra-specific variation in venom composition [7–15]. RNA amplification could be employed to increase the levels of RNA to those required for NGS, but this approach could introduce differential gene-amplification biases. Whilst acknowledging these limitations, it is also clear from the rapid technical advances in RNA-recovery kits and RNA-sequencing platforms that it will not be long before RNA recovered from TRIzol-treated venom samples will be the norm to ethically produce accurate venom transcriptomics—for whatever biological purpose and from wherever the samples are collected.

## Supporting Information

S1 Table Primers for elapid PLA_2_ (A), KSPI (B), CTL (C) and SVMP (D). (PDF)

S1 Fig Multiple sequence alignments for PLA2 (A), KSPI (B), CTL (C) and SVMP (D) amino acid sequences translated from transcripts isolated from the V^TRZ^ of several Elapidae species: *A*. *s*. *intermedius*, *A*. *l*. *cowlesi*, *A*. *l*. *lubricus* and *N*. *kaouthia*. (PDF)
