# Supplementary material for: Stabilising the Integrity of Snake Venom mRNA Stored under Tropical Field Conditions Expands Research Horizons
Source: PLoS Negl Trop Dis. 2016 Jun 9;10(6):e0004615. doi: 10.1371/journal.pntd.0004615 (PMC4900621; doi:10.1371/journal.pntd.0004615)
Supplement: S1 Table — (PDF) [file pntd.0004615.s001.pdf]

S1 Table: Primers for Elapidae PLA_2 (A), KSPI (B), CTL (C) and SVMP (D)

(A) Group 1 phospholipase A2

List of forward primers	List of reverse primers
AGCTTCACCACGGACAGATG	CACTGAAGCCTCTCAAATATCA
TTCACCACGGACAGATG	AGGTTAGTAACTGCCACCGTC
CAGCTTCACCAGGGACAAAAT	TTGCGCTGAAGCCTCTCAAATAT
ACTCATCTTGCTTGCAGCTTCA	TAATAGAGGCCCGTCCGA
GCAGTTTGTGTCTCCCTCTTA	GATTGCCTCTCAAATATC
	AGAGAATTGCCACGTGCAGGTG
	TGCCACAGTCCTTGCGC
	AATAGAGGCCCGTCCAGAG
	CCTTCTGTTTCACGGGCA
	ATCCAGAGAATTGCCACGT
	CACTTTATTGTTCAGGAAACGG

(B) C-type lectins

List of forward primers	List of reverse primers
ACAGGGAAGGAAGGAAGA	GGTGAGCAAAATCAGACC
AAGGAAGGAAAGAAGACC	
TTGCCACTGAGAAGACTTTC	

(C) Kunitz-type serine protease inhibitors

List of forward primers	List of reverse primers
AGAGAGAGAGCTTCATCATGT	AAACAAAGGAATGAGCCC

(D) Snake venom metalloproteinases

List of forward primers	List of reverse primers
AGAGCCAGTGGTCCTTCAC	TCCCATTGTAGCAGTAAC
