# Supplementary material for: Stabilising the Integrity of Snake Venom mRNA Stored under Tropical Field Conditions Expands Research Horizons
Source: PLoS Negl Trop Dis. 2016 Jun 9;10(6):e0004615. doi: 10.1371/journal.pntd.0004615 (PMC4900621; doi:10.1371/journal.pntd.0004615)
Supplement: S1 Fig — (PDF) [file pntd.0004615.s002.pdf]

S1 Fig: Multiple sequence alignments for PLA_2 (A), KSPI (B), CTL (C) and SVMP (D) amino acid sequences translated from transcripts isolated from the V^{TRZ} of several Elapidae species: *A. s. intermedius*, *A. l. cowlesi*, *A. l. lubricus* and *N. kaouthia*

(A) Group 1 phospholipase A_2

```
Elapid_Venom_PLA2_01   1 ?NPAQLLVLAAVYVSPLGAASIRPMPLNLLQFNNMIQCTIPTSIPWLDYSNYGCYCGYGG
Elapid_Venom_PLA2_02   1 ?NPAQLLVLAAVYVSPLGAASIRPMPLNLLQFNNMIQCTIPTSIPWLDYSNYGCYCGYGG
Elapid_Venom_PLA2_03   1 ?NPAQLLVLAAACVSPLGTASVRPLPLNLLQFNSMIQCTIPTSIPWLDYSNYGCYCGYGG
Elapid_Venom_PLA2_04   1 ?NPAQLLVLAAVCVSPLGTASVRPLPLNLLQFNNMIQCTIPTSIPWLDYSNYGCYCGYGG
Elapid_Venom_PLA2_05   1 ?NPAQLLVLAAVCVSPLGTASVRPLPLNLLQFNNMIQCIIPTSIPWLDYSNYGCYCGYGG
Elapid_Venom_PLA2_06   1 ?NPAQLLVLAAVCVSPLGAASIRPMPLNLLQFNNMIQCTIPTSIPWSDYSNYGCYCGYGG
Elapid_Venom_PLA2_07   1 ?NPAQLLVLAAVCVSPLGAASIRPMPLNLLQFNNMIQCTIPTSIPWSDYSNYGCYCGYGG
Elapid_Venom_PLA2_08   1 ?NPAQLLVLAAVCVSPLGAASIRPMPLNLLQFNNMIQCTIPTSIPWLDYSNYGCYCGYGG
Elapid_Venom_PLA2_09   1 ?NPAQLLVLAAVCVSPLGAASIRPMPLNLLQFNNMIQCTIPTSIPWLDYSNYGCYCGYGG
Elapid_Venom_PLA2_10   1 ?NPAQLLVLAAVCVSPLGAASIRPMPLNLLQFNNMIQCTVPTSIPWLDYSNYGCYCGYGG
Elapid_Venom_PLA2_11   1 ?NPAQLLVLAAVCVSPLGAASIRPMPLNLLQFNNMIQCTIPTSIPWLDYSNYGCYCGYGG
Elapid_Venom_PLA2_12   1 ?NPAQLLVLAAVCVSPLGAASVRPLPLNLLQFNNMIQCTIPTSIPWLDYSNYGCYCGYGG
Elapid_Venom_PLA2_13   1 MNPAQLLVLAAVCVSPLGAASIRPMPLNLLQFNNMIQCTIPTSIPWLDYSNYGCYCGYGG
Elapid_Venom_PLA2_14   1 ?NPAQLLVLAAVCVSPLGAASVRPLPLNLLQFNNMIQCTISTSIPWLDYSNYGCYCGYGG
Elapid_Venom_PLA2_15   1 ?NPAQLLVLAAVCVSPLGAASIRPMPLNLLQFNNMIQCTIPTSIPWLDYSNYGCYCGYGG
Elapid_Venom_PLA2_16   1 ?NPAQLLVPAAVCVSPLGAASIRPMPLNLLQFNNMIQCTIPTSIPWLDYSNYGCYCGYGG
Elapid_Venom_PLA2_17   1 MNPAQLLVLAAVCVSPLGAASIRPMPLNLLQFNNMIQCTIPTSIPWLDYSNYGCYCGYGG
Elapid_Venom_PLA2_18   1 ?NPAQLLVLAAVCVSPLGAASIRPMPLNLLQFNNMIQCTIPTSIPWLDYSNYGCYCGYGG
Elapid_Venom_PLA2_19   1 ?NPAQLLVLAAVCVSPLGAASIRPMPLNLLQFNNMIQCTIPTSIPWLDYSNYGCYCGYGG
Elapid_Venom_PLA2_20   1 ?NPAQLLVLAAVCVSPLGAASIRPMPLNLLQFNNMIQCTIPTSIPWLDYSNYGCYCGYGG
Elapid_Venom_PLA2_21   1 ?NPAQLLVLVAVCVSPLGAASIRPMPLNLLQFNNMIQCTIPTSIPWLDYSNYGCYCGYGG
Elapid_Venom_PLA2_22   1 ?NPAQLLVLAAVCVSPLGAASVRPLPLNLLQFNNMIQCTIPTSIPWLDYSNYGCYCGYGG
Elapid_Venom_PLA2_23   1 ?NPAQLLVLAAVCVSPLGAASIRPMPLNLLQFNNMIQCTIPTSIPWLDYSNYGCYCGYGG
Elapid_Venom_PLA2_24   1 ?NPAQLLVLAAVCVSPLGAASIRPMPLNLLQFNNMIQRTIPTSIPRLDYSNYGCYCGYGG
Elapid_Venom_PLA2_25   1 ?NPAQLLVLAAVCVSPLGAASIRPMPLNLLQFNNMIQCTIPTSIPWLDYSNYGCYCGHGG
Elapid_Venom_PLA2_26   1 ?NPAQLLVLAAVCVSPLGAASIRPMPLNLLQFNNMIQCTIPTSIPWLDYSNYGCYCGYGG
Elapid_Venom_PLA2_27   1 ?NPAQLLVLAAVCVSPLGAASVRPLPLNLLQFNNMIRCTIPTSIPWLDYSNYGCYCGYGG
Elapid_Venom_PLA2_28   1 MNPAQLLVLAAVCVSPLGAASVRPLPLNLLQFNNVIQCTIPTSIPWLDYSSYGCYCGYGG
Elapid_Venom_PLA2_29   1 MNPAQLLVLAAVCVSPLGAASVRPLPLNPLQFNNMIQCTIPTSIPWLDYSNYGCYCGYGG
Elapid_Venom_PLA2_30   1 ?????????????????GAASIRPMPLNPLQFNNMIQCTIPTSIPWLDYSNYGCYCGYGG
Elapid_Venom_PLA2_31   1 ?????????????????????????PLNPLQFNNMIQCTIPTSIPWLDYSNYGCYCGYGG
Elapid_Venom_PLA2_32   1 ?NPAQLLVLAAVCVSPLGAASIRPMPLNLLQFNNMIQCTIPTSIPWLDYSNYGCYCGYGG
Elapid_Venom_PLA2_33   1 ?NPAQLLVLAAVCVSPLGAASVRPLPLNLLQFNNMIQCTIPTSIPWLDYSNYGCYCGYGG
Elapid_Venom_PLA2_34   1 ?????????????????GAASVRPLPLNLLQFNNMIQCTIPTSIPWLDYSNYGCYCGYGG
Elapid_Venom_PLA2_35   1 MNPAQLLVLAAVCVSPLGAASIRPMPLNLLQFNNMIQCTIPTSIPWLDYSNYGCYCGYGG
Elapid_Venom_PLA2_36   1 ?NPAQLLVLAAVCVSPLGAASIRPMPLNLLQFNNMIQCTIPTSIPWLDYSNYGCYCGYGG
Elapid_Venom_PLA2_37   1 ??????????AVCVSLLGAASIRPMPLNLLQFNNMIQCTIPTSIPWLDYSNYGCYCGYGG
Elapid_Venom_PLA2_38   1 ?????????????????GAASIRPMPLNLLQFNNMIQCTIPTSIPWLDYSNYGCYCGYGG
Elapid_Venom_PLA2_39   1 ?????????????????GAASIRPMPLNLLQFNNMIQCTIPTSIPWLDYSNYGCYCGYGG
Elapid_Venom_PLA2_40   1 ?????????????????GAASIRPMPLNLLQFNNMIQCTIPTSIPWLDYSNYGCYCGYGG
Elapid_Venom_PLA2_41   1 ?????????????????GAASIRPMPLYLLQFNNMIQCTIPTSIPWLDYSNYGCYCGYGG
Elapid_Venom_PLA2_42   1 ?????????????????GAASIRPMPLNLIQFNNMIQCTIPTSIPWLDYSNYGCYCGYGG
Elapid_Venom_PLA2_43   1 ?????????????????GAASIRPMPLNLLQFNNMIQCTIPTSIPWLDYSNYGCYCGYGG
Elapid_Venom_PLA2_44   1 ?????????????????GAASICPMPLNLLQFNNMIQCTIPTSIPWLDYSNYGCYCGYGG
Elapid_Venom_PLA2_45   1 ?????????????????GAASIRPMPLNLLQFNNMIQCTIPTSIPWLDYSNYGCYCGYGG
Elapid_Venom_PLA2_46   1 ?????????????????GAASIRPMPLNLLQFNNLIQCTIPTSIPWLDYSNYGCYCGYGG
Elapid_Venom_PLA2_47   1 ?????????????????GAASVRPLPLNLLQFNNMIQCTIPTSIPWLDYSNYGCYCDYGG
Elapid_Venom_PLA2_48   1 ?????????????????GAASVRPLPLNLLQFSNMIQCTIPTSIPWLDYSNYGCYCGYGG
Elapid_Venom_PLA2_49   1 ?????????????????GAASVRPLPLNLLQFNNMIQCTIPTSIPWLDYSNYGCYCGYGG
Elapid_Venom_PLA2_50   1 ?????????????????GAASVRPLPLNLLQFNNMIQCTIPTSIPWLDYSNYGCYCGYGG
Elapid_Venom_PLA2_51   1 ?????????????????GAASVRPLPLNLLQFNNMIQCTIPTSIPWLDYSNYGCYCGYGG
Elapid_Venom_PLA2_52   1 ?????????????????GAASVRPLPLNLLQFNNMIQCTIPTSIPWLDYSNYGCYCGYGG
Elapid_Venom_PLA2_53   1 ?????????????????GAASVRPLPLNLLQFNNMIQCTIPTSIPWLDYSNYGCYCGYGG
Elapid_Venom_PLA2_54   1 ?????????????????GAASVRPLPLNLLQFNNMIQCTIPTSIPWLDYSNYGCYCGYGG
Elapid_Venom_PLA2_55   1 ?????????????????GAASVRPLPLNLLQFNNMIQCTIPTSIPWLDYSNYGCYCGYGG
Elapid_Venom_PLA2_56   1 ?????????????????GAASVRPLPLNLLQFNNMIQCTIPTSIPWLDYSNYGCYCGYGG
Elapid_Venom_PLA2_57   1 ?????????????????GAASVRPLPLNLLQFNNMIQCTIPTSIPWLDYSNYGCYCGYGG
Elapid_Venom_PLA2_58   1 ?????????????????GAASVRPLPLNLLQFNNMIQCTIPTSIPWLDYSNYGCYCGYGG
```

```
Elapid_Venom_PLA2_59    1 ?????????????????GAASVRPLPLNLLQFNYMIQCTIPTSIPWLDYSNYGCYCGYGG
Elapid_Venom_PLA2_60    1 ??????????????????????RPLPLNLLQFNYMIQCTIPTSIPWLDYSNYGCYCGYGG
Elapid_Venom_PLA2_61    1 ?????????????????????????PLNLLQFNNMIQCTIPTSIPWLDYSNYGCYCGYGG
Elapid_Venom_PLA2_62    1 ?????????????????????????PLNLLQFNNMIQCTIPTSIPWLDYSNYGCYCGYGG
Elapid_Venom_PLA2_63    1 ?????????????????????????PLNLLQFNNMIQCTIPTSIPWLDYSNYGCYCGYGG
Elapid_Venom_PLA2_64    1 ?????????????????????????PLNLLQFNNMIQCTIPTSIPWLDYSNYGCYCGYGG
Elapid_Venom_PLA2_65    1 ?NPAQLLVLAAVCVSPLGAASIRPMPLNLLQFNNMIQCTIPTSIPWLDYSNYGCYCGYGG
Elapid_Venom_PLA2_66    1 MNPAQLLVLAAVCVSPLGAASIRPMPLNLLQFNNMIQCTIPTSIPWLDYSNYGCYCGYGG
Elapid_Venom_PLA2_67    1 ?NPAQLLVLAAVCVSPLGAASIRPMPLNLLQFNNMIQCTIPTSIPWLDYSNYGCYCGYGG
Elapid_Venom_PLA2_68    1 ?NPAQLLVLAAVCVSLLGASSIRPMPLNLYQFKNMIKCTVPSRS-WWDFADYGCFCGYGG
Elapid_Venom_PLA2_69    1 ?NPAQLLVLAAVCVSLLGASSIRPMPLNRYQFKNMIKCTVPSRS-WWDFADYGCFCGYGG
Elapid_Venom_PLA2_70    1 ?????????????????GASSTRPMPLNLYQFKNTIKCTVPSRS-WWDFADYGCFCGYGG
Elapid_Venom_PLA2_71    1 ?NPAQLLVLAAVCVSLLGASSIRPMPLNLYQFKNMIKCTVPSRS-WWDFADYGCFCGYGG
Elapid_Venom_PLA2_72    1 ?NTAKLLVLAAVCVSLLGASSIRPMPLNLYQFKNMIKCTVPSRS-WWDFADYGCFCGYGG
Elapid_Venom_PLA2_73    1 MNPAQLLVLAAVCVSLLGASSIRPMPLNLYQFKNMIKCTVPSRS-WWDFADYGCFCGYGG
Elapid_Venom_PLA2_74    1 MNPAQLLVLAAVCVSLLGASSIRPMSLNLYQFKNMIKCTVPSRS-WWDFADYGCFCGYGG
Elapid_Venom_PLA2_75    1 ?NPAQLLVLAAVCVSLFGASSIRPMPLNLYQFKNMIKCTVPSRS-WWDFADYGCFCGYGG
Elapid_Venom_PLA2_76    1 ?NPAQLLVLTAVCVSLLGASSIRPMPLNLYQFKNMIKCTVPSRS-WWDFADYGCFCGYGG
Elapid_Venom_PLA2_77    1 ?NPAQLLVLAAVCVSLLGASSIRPTPLNLYQFKNMIKCTVPSRS-WWDFADYGCFCGYGG
Elapid_Venom_PLA2_78    1 MNPAQLLVLAAVCVSLLGASSVRPMPLNLYQFKNMIKCTVPSRS-WWDFTDYGCFCGYGG
Elapid_Venom_PLA2_79    1 MNPAQLLVLAAVCVSLLGASSIRPMPLNLYQFKNMIKCTVPSRS-WWDFADYGCFCGYGG
Elapid_Venom_PLA2_80    1 ???????????VCVSLLGASSIRPMPLNLYQFKNMIKCTVPSRS-WWDSADYGCFCGYGG
Elapid_Venom_PLA2_81    1 ?????????????????GASSIRPMPLNLYQFKNMIKCTVPSRS-WWDFADYGCFCGYGG
Elapid_Venom_PLA2_82    1 MNPAQLLVLAAVCVSLLGASSIRPMPLNLYQFKNMIKCTVPSRS-WWDFADYGCFCGYGG
Elapid_Venom_PLA2_83    1 ?NPAQLLVLAAVCVSLLGASSIRPMPLNLYQFKNMIKCTVPSRS-WWDFADYGCFCGYGG
Elapid_Venom_PLA2_84    1 ?NPAQLLVLAAVCVPLLGASSIRPMPLNLYQFKNMIKCTVPSRS-WWDFADYGCFCGYGG
Elapid_Venom_PLA2_85    1 ?NPAQLLVLAAVCVSLLGASSIRPMPLNLYQFKNMIKCTVPSRS-WWDFADYGCFCGYGG
Elapid_Venom_PLA2_86    1 ????????????CVSLLGASSIRPMPLNLYQFKDMIKCTVPSRS-WWDFADYGCFCGYGG
Elapid_Venom_PLA2_87    1 ?????????????????GASSIRPMPLNLYQFKNMIKCTVPSRS-WWDFADYGCFCGYGG
Elapid_Venom_PLA2_88    1 ??????????????????????????????????????????RS-WWDFADYSCFCGYGG
Elapid_Venom_PLA2_89    1 MNPAQLLVLAAVCVSLLGASSIRPMPLNLYQFKNMIKCTVPSRS-WWDFADYGCFCGYGG
Elapid_Venom_PLA2_90    1 ?NPAQLLVLAAVCVSLLGASSIRPMPLNLYQFKNMIKCTVPSRS-WWDFADYGCFCGYGG
```

Elapid_Venom_PLA2_01	60	SGTPVDELDRCCQTHDNCYSEAEKLSECKPYYKIYSYECSEGTLTCGGDNEECAAFVCNC
Elapid_Venom_PLA2_02	60	SGTPVDELDRCCQTHDNCYSEAEKLSECKLYYKIYSYECSEGTLTCGGDNEECAAFVCNC
Elapid_Venom_PLA2_03	60	SGTPVDELDRCCQTHDNCYSEAEKLSECKPYFKIYSYECSEGTLTCGGDNEECAAFVCNC
Elapid_Venom_PLA2_04	60	SGTPVDELDRCCQTHDNCYSEAEKLSECKPYFKIYSYECSEGTLTCGGDNEECAAFVCNC
Elapid_Venom_PLA2_05	60	SGTPVDELDRCCQTHDNCYSEAEKLSECKPYFKIYSYECSEGTLTCGGDNEECAAFVCNC
Elapid_Venom_PLA2_06	60	SGTPVDELDRCCQTHDNCYSEAEKLSECKPYYKIYSYECSEGTLTCGGDNEECAAFVCNC
Elapid_Venom_PLA2_07	60	SGTPVDELDRCCQTHDNCYSEAEKLSECKPYYKIYSYECSEGTLTCGGDNEECAAFVCNC
Elapid_Venom_PLA2_08	60	SGTPVGELDRCCQTHDNCYPEAEKLSECKPYYKIYSYECSEGTLTCGGDNDECAAFVCNC
Elapid_Venom_PLA2_09	60	SGTPVDELDRCCQTHDNCYSEAEKLSECKPYYKICSYECSEGTLTCGGDNDECAAFVCNC
Elapid_Venom_PLA2_10	60	SGTPVDELDRCCQTHDNCYSEAEKLSECKPYYKIYSYECSEGTLTCGGDNDECAAFVRNC
Elapid_Venom_PLA2_11	60	SGTPVDELDRCCQTHDNCYSEAEKLSECKPYYKIYSYECSEGTLTCGGDNDECAAVVCNC
Elapid_Venom_PLA2_12	60	SGTPVDELDRCCQTHDNCYSEAEKLSECKPYYKIYSYECSEGTLTCGGDNEECAAFVCNC
Elapid_Venom_PLA2_13	61	SGTPVDELDRCCQTHDNCYSEAEKLSECKPYYKIYSYECSEGTLTCGGDNEECAAFVCNC
Elapid_Venom_PLA2_14	60	SGTPVDELDRCCQTHDNCYSEAEKLSECKPYYKIYSYECSEGTLTCGGDNEECAAFVCNC
Elapid_Venom_PLA2_15	60	SGTPVDELDRCCQTHDNCYSEAEKLSECKPYYKIYSYECSEGTLTCGGDNDECAAFVCNC
Elapid_Venom_PLA2_16	60	SGTPVDELDRCCQTHDNCYSEAEKLSECKPYYKIYSYECSEGALTCGGDNDECAAFVCNC
Elapid_Venom_PLA2_17	61	SGTPVDELDRCCQTHDNCYSEAEKLSECKPYYKIYSYECSEGTLACGGDNDECAAFVCNC
Elapid_Venom_PLA2_18	60	SGTPVDELDRCCQTHVNCYSEAEKLSECKPYYKIYSYECSEGTLTCGGDNDECAAFVCNC
Elapid_Venom_PLA2_19	60	SGTPVDELDRCCQTHDNCYSEAEKLSECKLYYKIYSYECSEGTLTCGGDNDECAAFVCNC
Elapid_Venom_PLA2_20	60	SGTPVDELDRCCQTHDNCYSEAEKLSECKPYYKIYPYECSEGTLTCGGDNEECAAFVCNC
Elapid_Venom_PLA2_21	60	SGTPVDELDRCCQTHDNCYSEAEKLSECKPYYKIYSYECSEGTLTCGGDNEECAAFVCNC
Elapid_Venom_PLA2_22	60	SGIPVDELDRCCQTHDNCYSEAEKLSECKPYYKIYSYECSEGTLTCGGDNEECAAFVCNC
Elapid_Venom_PLA2_23	60	SGTPVDELDRCCQTHDNCYSEAEKLSECKPYYEIYSYECSEGTLTCGGDDDECAAFVCNC
Elapid_Venom_PLA2_24	60	SGTPVDELDRCCQTHDNCYPEAEKLSERKPYYEIYSYECSEGTLTCGGDNDECAAFVCNC
Elapid_Venom_PLA2_25	60	SGTPVDELDRCCQTHDNCYSEAEKLSECKPYYKIYSYECSEGTLTCGGDNDECAAFVCNC
Elapid_Venom_PLA2_26	60	SGTPVDELDRCCQTHDNCYSEAEKLSECKPYYKIYSYECSEGTLTCGGDNDECAAFVCNC
Elapid_Venom_PLA2_27	60	SGTPIDELDRCCQTHDNCYSEAEKLSECKPYYKIYSYECSEGTLTCGGDNEECAAFVCNC
Elapid_Venom_PLA2_28	61	SGTPVDELGRCCQTHDNCYSEAEKLSECKPYYKIYSYECSEGTLTCGGDNEECAAFVCNC
Elapid_Venom_PLA2_29	61	SGTPVDELDRCCQTHDNCYSEAEKLSECKPYYKIYSYECSEGTLTCGGDNEECAAFVCNC
Elapid_Venom_PLA2_30	44	SGTPVDELDRCCQTHDNCYSEAEKLSECKPYYKIYSYECSEGTLTCGGDNEECAAFVCNC
Elapid_Venom_PLA2_31	36	SGTPVDELDRCSQTHDNCYSEAEKLSECKPYYKIYSYGCSEGTLTCGGDNEECAAFVCNC
Elapid_Venom_PLA2_32	60	SGTPVDELDRCCQTHDNCYSEAEKLSECKPYYKIYSYECSEGTLTCGGDNDECAAFVCNC
Elapid_Venom_PLA2_33	60	SGTPVDELDRCCQTHDNCYSEAEKPSECKPYYKIYSYECSEGTLTCGGDNEECAAFVCNC
Elapid_Venom_PLA2_34	44	SGTPVDELDRCCQTHDNCYSEAEKRSECKPYYKIYSYECSEGTLTCGGDNEECAAFVCNC
Elapid_Venom_PLA2_35	61	SGTPVDELDRCCQTHDNCYSEAEKLSECRPYYKIYSYECSEGTLTCGGDNEECAAFVCNC
Elapid_Venom_PLA2_36	60	SGTPVDELDRCCQTHDNCYSEAEKLSECKPYYKIYSYECSEGTLTCGGDNEECAAFVCNC
Elapid_Venom_PLA2_37	51	SGTPVDELDRCCQTHDNCYSEAEKLSECKPYYKIYSYECSEGTLTCGGDNEECAAFVCNC
Elapid_Venom_PLA2_38	44	SGTPVDELDRCCQTHDNCYSEAEKLSECKPYYKIYSYECSEGTLTCGGDNEECAAFVCNC
Elapid_Venom_PLA2_39	44	SGTPVDELDRCCQTHDNCYSEAEKLSECKPYYKIYSYECSEGTLTCGGDNEECATFVCNC
Elapid_Venom_PLA2_40	44	SGTPVDELDRCCQTHDNCYSEAEKLSECKPYYKIYSYECSEGTLTCGGDNEECAAFVSNC
Elapid_Venom_PLA2_41	44	SGTPVDELDRCCQTHDNCYSEAEKLSECKPYYKIYSYECSEGTLTCGGDNEECAAFVCNC
Elapid_Venom_PLA2_42	44	SGTPVDELDRCCQTHDNCYSEAEKLSECKPYYKIYSYECSEGTLTCGGDNEECAAFVCNC
Elapid_Venom_PLA2_43	44	SGTPVDELDRCCQTHDNCYSEAEKLSECKPYYKIYSYECSEGTLTCGGDNEECAALVCNC
Elapid_Venom_PLA2_44	44	SGTPVDELDRCCQTHDSCYSEAEKLSECKPYYKIYSYECSEGTLTCGGDNEECAAFVCNC
Elapid_Venom_PLA2_45	44	SGTPVDELDRCCQTHDNCYSEAEKLSECKPYYKIYSYECSEGTLTCGGDNEECAAFVCNC
Elapid_Venom_PLA2_46	44	SGTPVDELDRCCQTHDNCYSEAEKLSECKPYYKIYSYECSEGTLTCGGDNEECAAFVCNC
Elapid_Venom_PLA2_47	44	SGTPVDELDRCCQTHDNCYSEAEKLSECKPYYKIYSYECSEGTLTCGGDNDECAAFVCNC
Elapid_Venom_PLA2_48	44	SGTPVDELDRCCQTHDNCYSEAEKLSECKPYYKIYSYECSEGTLTCGGDNEECAAFVCNC
Elapid_Venom_PLA2_49	44	SGTPVDELDRCCQTHDNCYSEAEKLSECKPYYKIYSYECSEGTLTCGGDNEECAAFVCNC
Elapid_Venom_PLA2_50	44	SGTPVDELDRCCQTHDNCYSEAEKLSECKPYYKIYSYECSEGTLTCGGDNEECAAFVCNC
Elapid_Venom_PLA2_51	44	SGTPVDELDRCCQTHDNCYSEAEKLSECKPYYKIYSYECSEGTLTCGGDNEECAAFVCNC
Elapid_Venom_PLA2_52	44	SGTPVDELDRCCQAHDNCYSEAEKLSECKPYYKIYSYECSEGTLTCGGDNEECAAFVCNC
Elapid_Venom_PLA2_53	44	SGTPVDELDRCCQTHDNCYSEAEKLSECKPYYKIYSYECSEGTLTCGGDSEECAAFVCNC
Elapid_Venom_PLA2_54	44	SGTPVDELDRCCQTHDNCYSGAEKLSECKPYYKIYSYECSEGTLTCGGDNEECAAFVCNC
Elapid_Venom_PLA2_55	44	SGTPVDELDRCCQTHDNCYSEAEKLSECKPYYKIYSYECSEGTLTCGGDNEECAAFVCNC
Elapid_Venom_PLA2_56	44	SGTPVDELDGCCQTHDNCYSEAEKLSECKPYYKIYSYECSEGTLTCGGDNEECAAFVCNC
Elapid_Venom_PLA2_57	44	SGTPVDELDRCCQTHDNCYSEAEKLSECKPHYKIYSYECSEGTLTCGGDNEECAAFVRNC
Elapid_Venom_PLA2_58	44	SGTPVDELDRCCQTHDNCYSEAEKLSECKPYYKIYSYECSEGTLTCGGDNKECAAFVCNC
Elapid_Venom_PLA2_59	44	SGTPVDELDRCCQTHDNCYSEAEKLSECKPYYKIYSYECSEGTLTCGGDNEECAAFVCNC
Elapid_Venom_PLA2_60	39	SGTPVDELDRCCQTHDNCYSEAEKLSECKPYYKIYSYECSEGTLTCGGDNEECAAFVCNC
Elapid_Venom_PLA2_61	36	SGTPVDELDRCCQTHDNCYPEAEKLSECEPYYKIYSYECSEGTLTCGGDNEECAAFVCNC
Elapid_Venom_PLA2_62	36	SGTPVDELDRCCQTHDNCYSEAEKLSECEPYYKIYSYECSEGTLTCGGDNEECTAFVCNC
Elapid_Venom_PLA2_63	36	SGTPVDELVRCCQTHDNCYSEAENLSECKPYYKIYSYECSEGTLTCGGDNEECAAFVCNC
Elapid_Venom_PLA2_64	36	SGTPVDELDRCCQTHDNCYSEAEKLSECKPYYKIYSYECSEGTLTCGGDNEECAAFVCNC
Elapid_Venom_PLA2_65	60	SGTPVDDLDRCCQTHDNCYSEAEKLPSCTPYIKTYSYECSEGTLTCGGDNDECAAFVCNC
Elapid_Venom_PLA2_66	61	SGTPVDELDRCCQTHDNCYSEAEKLSECKPYYKIYSYECSEGTLTCGGDNEECAAFVCNC

```
Elapid_Venom_PLA2_67    60 SGTPVDDLDRCCQTHDNCYSEAEKLPSCTAYIKTYSYECSEGTLTCGGGNDECAAFVCNC
Elapid_Venom_PLA2_68    59 SGTPVDDLDRCCQTHDNCYSEAEKLPSCTPYIKTYSYECSEGTLTCGGGNDECAAFVCNC
Elapid_Venom_PLA2_69    59 SGTPVDDLDRCCQTHDSCYSEAEKLPSCTPYIKTYSYECSEGTLTCGGGNDECAAFVCNC
Elapid_Venom_PLA2_70    43 SGTPVDDLDRCCQTHDNCYSEAEKLPSCTPYIKTYSYECSEGTLTCGGDNDECAAFVCNC
Elapid_Venom_PLA2_71    59 SGTPVDDLDRCCQTHDNCYSEAEKLPSCTPHIKTYSYECSEGTLTCGGDNDECAAFVCNC
Elapid_Venom_PLA2_72    59 SGTPVDDLDRCCQTHDNCYSGAEKLPSCTPYIKTYSYECSEGTLTCGGDNDECAAFVCNC
Elapid_Venom_PLA2_73    60 SGTPVDDLDRCCQTHDNCYSGAEKLPSCTPYIKTYSYECSEGTLTCGGDNDECAAFVCNC
Elapid_Venom_PLA2_74    60 SGTPVDDLDRCCQTHDNCYSEAEKLPSCTPYIKTYSYECS????????????????????
Elapid_Venom_PLA2_75    59 SGTPVDDLDRCCQTHDNCYSEAEKLPSCTPYVKTYSYECSEGTLTCGGDNDECAAFVCNC
Elapid_Venom_PLA2_76    59 SGTPVDDLDRCCQTHDNCYSEAEKLPSCTPYIKTYSYECSEGTLTCGGDNDECAAFVCNC
Elapid_Venom_PLA2_77    59 SGTPVDDLDRCCQTHDNCYSEAEKLPSCTPYIKTYPYECSEGTLTCGGDNDECAAFVRNC
Elapid_Venom_PLA2_78    60 SGTPVDDLDRCCQTHDNCYSEAEKLPSCTPYIKTYSYECSEGTLTCGGDNDECAAFVCNC
Elapid_Venom_PLA2_79    60 SGTPVDDLDRCCQTHDNCYSEAEKLPSCTPYIKTYSYECSAGTLTCGGDNDECAAFVCNC
Elapid_Venom_PLA2_80    49 SGTPVDDLDRCCQTHDNCYSEAEKLPSCTPYIKTYSYECSAGTLTCGGDNDECAAFVCNC
Elapid_Venom_PLA2_81    43 SGTPVDDLDRCCQTHDNCYSEAEKLPSCTPYIKTYSYECSAGTLTCGGDNDECAAFVCNC
Elapid_Venom_PLA2_82    60 SGTPVDDLDRCCQTHDNCYSEAEKLPSCTPYIKTYSYECSEGTLTCGGDNDECAAFVCNC
Elapid_Venom_PLA2_83    59 SGTPVDDLDRCCQTHDNCYSEAEKLPSCTPYIKTYSYECFEGTLTCGGDNDECAAFVCNC
Elapid_Venom_PLA2_84    59 SGTPVDDLDRCCQTHDNCYSEAEKLPSCTPYIKTYSYECSEGTLTCGGDNDECAAFVCNC
Elapid_Venom_PLA2_85    59 SGTPVDDLDRCCQTHDNCYSEAEKLPSCTPYIKTYSYECSTGTLTCGGDNDECAAFVCNC
Elapid_Venom_PLA2_86    48 SGTPVDDLDRCCQTHDNCYSEAEKLPSCTPYIKTYSYECSEGTLTCGGDDDECAAFVCNC
Elapid_Venom_PLA2_87    43 SGTPVDDLDRCCQTHDNCYSEAEKLPSCTPYIKTYSYECSEGTLTCGGDNDECAALVCNC
Elapid_Venom_PLA2_88    18 SGTPVDDLDRCCQTHDNCYSEAEKLPSCTPYIKTYSYECSEGTLTCGGDNDECAAFVCNC
Elapid_Venom_PLA2_89    60 SGTPVDDLDRCCQTHDNCYSEAEKLPSCTPYIKTYSYECSEGTLTCGGDNDECAAFVCNC
Elapid_Venom_PLA2_90    59 SGTPVDDLDRCCQTHDDCYSVAEKLPSCTPYIKTYSYECSEGTLTCGGDNDECAAFVCNC
```

Elapid_Venom_PLA2_01	120	DREAAICFAGAPYIKANKKIDTKERCQ*
Elapid_Venom_PLA2_02	120	DREAAICFAGAPYIKANKKIDTKERCQ?
Elapid_Venom_PLA2_03	120	DREAAICFAGAPYIKANKKIDTKERCQ*
Elapid_Venom_PLA2_04	120	DREAAICFAGAPYIKANKKIDTKERCQ*
Elapid_Venom_PLA2_05	120	DREAAICFAGAPYIKANKKIDAKERCQ*
Elapid_Venom_PLA2_06	120	DREAAICFAGAPYIRANKKIDTKERCQ*
Elapid_Venom_PLA2_07	120	DREAAICFAGAPYIKANKKIDTKERCQ*
Elapid_Venom_PLA2_08	120	DREAAICFAGAPYIKANKKIDTKERCQ*
Elapid_Venom_PLA2_09	120	DREAAICFAGAPYIKANKKIDTRERCQ*
Elapid_Venom_PLA2_10	120	DREAAICFAGAPYIKANKKIDTKERCQ?
Elapid_Venom_PLA2_11	120	DREAAICFAGAPYIKANKKIDTKERCQ*
Elapid_Venom_PLA2_12	120	DREAAICFAGAPYIKANKKIDTKERCQ*
Elapid_Venom_PLA2_13	121	DREAAICFAGAPYIKANKKIDTKERCQ*
Elapid_Venom_PLA2_14	120	DREAAICFAGAPYIKANKKIDTKERCQ*
Elapid_Venom_PLA2_15	120	DREAAICFAGAPYIKANKKTDTKERCQ*
Elapid_Venom_PLA2_16	120	DREAAICFAGAPYIKANKKIDTKERCQ*
Elapid_Venom_PLA2_17	121	DREAAICFAGAPYIKANKKIDTKERCQ*
Elapid_Venom_PLA2_18	120	DREAAICFAGAPYIKANKKIDTKERCQ*
Elapid_Venom_PLA2_19	120	DREAAICFAGAPYIKANKKIDTKERCQ*
Elapid_Venom_PLA2_20	120	DREAAICFAGAPYIKANKKIDTKERCQ*
Elapid_Venom_PLA2_21	120	DREAAICSAGAPYIKANKKIDTKERCQ*
Elapid_Venom_PLA2_22	120	DREAAICFAGAPYIKANKKIDTKERCQ*
Elapid_Venom_PLA2_23	120	DREAAICFAGAPYIKANKKIDTKERCQ*
Elapid_Venom_PLA2_24	120	DREAAICFAGAPYIKANKKIDTRERCQ*
Elapid_Venom_PLA2_25	120	DREAAICFAGAPYIKANKKIDTRERCQ*
Elapid_Venom_PLA2_26	120	DREAAICYAGAPYIKANKKIDTKERCQ*
Elapid_Venom_PLA2_27	120	DREAAICFAGAPYIKANKKIDTKERCQ?
Elapid_Venom_PLA2_28	121	DREAAICFAGAPYIKANKKIDTKERCQ*
Elapid_Venom_PLA2_29	121	DREATICFAGAPYIKANKKIDTKERCQ*
Elapid_Venom_PLA2_30	104	????????????????????????????
Elapid_Venom_PLA2_31	96	DREAAICFAGAPYIKANKKIDTKERCQ*
Elapid_Venom_PLA2_32	120	DREAAICFAGAPYIKANKKIDTKERCQ*
Elapid_Venom_PLA2_33	120	DREAAICFAGAPYIKANKKIDTKERCQ*
Elapid_Venom_PLA2_34	104	DREAAICFAGAPYIKANKKIDTKERCQ*
Elapid_Venom_PLA2_35	121	DREAAICFAGAPY???????????????
Elapid_Venom_PLA2_36	120	DHEAAICFAGAPY???????????????
Elapid_Venom_PLA2_37	111	DREAAICFAGAPYIKANKKIDTKERCQ*
Elapid_Venom_PLA2_38	104	DCEAAICFAGAPY???????????????
Elapid_Venom_PLA2_39	104	DREAAICFAGAPY???????????????
Elapid_Venom_PLA2_40	104	DREAAICFAGAPYIKANKKIDTKERCQ*
Elapid_Venom_PLA2_41	104	DREAAICFAGAPYIKANKKIDTKERCQ*
Elapid_Venom_PLA2_42	104	DREAAICFAGAPYIKANKKIDTKERCQ*
Elapid_Venom_PLA2_43	104	DREAAICFAGAPYIKANKKIDTKERCQ*
Elapid_Venom_PLA2_44	104	DREAAICFAGAPYIKANKKIDTKERCQ*
Elapid_Venom_PLA2_45	104	DRGAAICFAGAPYIKANKKIDTKERCQ*
Elapid_Venom_PLA2_46	104	DREAAICFAGAPYVKANKKIDTKERCQ*
Elapid_Venom_PLA2_47	104	DREAAICFAGAPYIKANKKIDTKERCQ*
Elapid_Venom_PLA2_48	104	DREAAICFAGAPYIKANKKIDTKERCQ*
Elapid_Venom_PLA2_49	104	DREAAICFAGAPYAKANKKIDTKERCQ?
Elapid_Venom_PLA2_50	104	DREAAICFAGAPYVKANKKIDTKERCQ?
Elapid_Venom_PLA2_51	104	DREAAICFAGAPYIKANRKIDTKERCQ?
Elapid_Venom_PLA2_52	104	DREAAICFAGAPYIKANKKIDTKERCQ*
Elapid_Venom_PLA2_53	104	DREAAICFAGAPYIKANKKIDTKERCQ*
Elapid_Venom_PLA2_54	104	DREAAICFAGAPYIKANKKIDTKERCQ?
Elapid_Venom_PLA2_55	104	DREAAICLAGAPYIKANKKIDTKERCQ?
Elapid_Venom_PLA2_56	104	DREAAICFAGAPYIKANKKIDTKERCQ?
Elapid_Venom_PLA2_57	104	DREAAICFAGAPYIKANKKIDTEERRQ?
Elapid_Venom_PLA2_58	104	DREAAICFAGAPYIKANKKIDTKERCQ?
Elapid_Venom_PLA2_59	104	DREAAICLAGAPYIKANKKIDTKERCQ?
Elapid_Venom_PLA2_60	99	DREAAICFAGAPYIKANKKIDTKERCQ?
Elapid_Venom_PLA2_61	96	DREAAICFAGAPYIKANKKIDTKERCQ?
Elapid_Venom_PLA2_62	96	DREAAICFAGAPYIKANKKIDTKERCQ?
Elapid_Venom_PLA2_63	96	DREAAICFAGAPYIKANKKIDTKERCQ*
Elapid_Venom_PLA2_64	96	DREATICFAGAPYIKANKKIDTKERCQ*
Elapid_Venom_PLA2_65	120	DRVAAICFAGAPYINDNYNVNLKERCQ*
Elapid_Venom_PLA2_66	121	DREAAICFAGAPYINDNYNVNLKERCQ*

```
Elapid_Venom_PLA2_67  120 DRVAAICFAGAPYINDNCNVNLKERCQ*
Elapid_Venom_PLA2_68  119 DRVAAICFAGAPYINDNCNVNLKERCQ*
Elapid_Venom_PLA2_69  119 DRVAAICFAGAPYINDNCNVNLKERCQ*
Elapid_Venom_PLA2_70  103 DRVAAICFAGAPYINDDYNVNLKERCQ*
Elapid_Venom_PLA2_71  119 DRVAAVRFAGAPYINDNYNVNLKERCQ*
Elapid_Venom_PLA2_72  119 DRVAAICFAGAPYINDNYNVNLKERCQ*
Elapid_Venom_PLA2_73  120 DRVAAICFAGAPYINDNYNVNLKERCQ*
Elapid_Venom_PLA2_74  100 ????????????????????????????
Elapid_Venom_PLA2_75  119 DRVAAICFAGAPYINDNYNVNLKERCQ?
Elapid_Venom_PLA2_76  119 DRVAAICFAGAPYINDNYNVNLKERCQ*
Elapid_Venom_PLA2_77  119 DRVAAICFAGAPYINDNYNVNLKERCQ*
Elapid_Venom_PLA2_78  120 DRVAAICFAGAPYINDSYNVNLKERCQ*
Elapid_Venom_PLA2_79  120 DRVAAICFAGAPYINDNYNVNLKERCQ*
Elapid_Venom_PLA2_80  109 DRVAAICFAGAPYINDNYNVNLKERCQ*
Elapid_Venom_PLA2_81  103 DRVAAVCFAGAPYINDNYNVNLKERCQ*
Elapid_Venom_PLA2_82  120 DRVAAICFAGAPYINDNYNVNLKERCQ*
Elapid_Venom_PLA2_83  119 DRVAAICFAGAPYINDNYNVNLKERCQ*
Elapid_Venom_PLA2_84  119 DRVAAICFAGAPYINDNYNVNLKERCQ*
Elapid_Venom_PLA2_85  119 DRVAAICFAGAPYINDN???????????
Elapid_Venom_PLA2_86  108 DRVAAICFAGAPYINDNYNVNLKERCQ*
Elapid_Venom_PLA2_87  103 DRVAAICFAGAPY???????????????
Elapid_Venom_PLA2_88   78 DRVAAICFAGAPYINDNYNVNLKERCQ*
Elapid_Venom_PLA2_89  120 DRVAAICFAGAPYINNNYNVNLKERCQ?
Elapid_Venom_PLA2_90  119 DRVAAICFAGAPYINNNYNVNLKERCQ?
```

(B) Kunitz-type serine protease inhibitors

```
Elapid_Venom_KSPI_01     1 SGHLLLLLGLLTLWAELTPVSGLGGPEYCLLPADPGPCSNYRYVYYYNPALRKCEQFLYG
Elapid_Venom_KSPI_02     1 SGHLLLLLGLLTLWAELTPVSGLGGPEYCLLPADPGPCSNYRYVYYYNPALRKCEQFLYG
Elapid_Venom_KSPI_03     1 SGHLLLLLGLLTLWAELTPVSGR--PGLCELPAASGLCNANIPAFYYNLAAKQCQKFIYG
Elapid_Venom_KSPI_04     1 SGHLLLLLGLLTLWAELTPVSGR--PGLCELPAASGLCNANIPAFHYNLAAKQCQKFIYG
Elapid_Venom_KSPI_05     1 SGHLLLQLGLLTLWAELTPVSGR--PGLCELPAASGLCNANIPAFYYNLAAKQCQKFIYG
Elapid_Venom_KSPI_06     1 SGHLLLLLGLLTLWAELTPVSGR--PGLCELPAASGLCNTNIPAFYYNLAAKQCQKFIYG
Elapid_Venom_KSPI_07     1 SGHLLLLLGLLTLWAELTPVSGR--PGLCELPAASGLCNTNIPAFYYNLAAKQCQKFIYG
Elapid_Venom_KSPI_08     1 SGHLLLLLGLLTLWAELTPVSGR--PGLCELPAASGLCNANIPAFYYNLAAKQCQKFIYG
Elapid_Venom_KSPI_09     1 SGHLLLLLGLLTLWAELTPASGR--PGLCELPAASGLCNANIPAFYYNLAAKQCQKFIYG
Elapid_Venom_KSPI_10     1 SGHLLLLLGLLTLWAELTPVSGR--PGLCELPAASGLCNANIPAFYYNLAAKQCQKFIYG
Elapid_Venom_KSPI_11     1 SGHLLLLLGLLTLWAELTPVSGR--PGLCELPAASGLCNANIPAFYYNLAAKQCQKFIYG
Elapid_Venom_KSPI_12     1 SGHLLLPLGLLTLWAELTPVSGR--PGLCELPAASGLCNANIPAFYYNLAAKQCQKFIYG
Elapid_Venom_KSPI_13     1 PGHLLLLLGLLTLWAELTPVSGR--PGLCELPAASGLCNANIPAFYYNLAAKQCQKFIYG
Elapid_Venom_KSPI_14     1 SGHLLLLLGLLTLWAELTPVSSR--PGLCELPAASGLCNANIPAFYYNLAAKQCQKFIYG
Elapid_Venom_KSPI_15     1 SGHLLLLLGLLTLWAELTPVSGR--PGLCELPAASGLCNANIPAFYYNLAAKQCQKFIYG
Elapid_Venom_KSPI_16     1 ?????????????????TPVSGR--PGLCELPAASGLCNANIPAFYYNLAAKQCQKFIYG

Elapid_Venom_KSPI_01    61 GCEGNKNNFKTRHECHRACVR?
Elapid_Venom_KSPI_02    61 GCEGNKNNFKTIDECRRTCAG?
Elapid_Venom_KSPI_03    59 GCGGNANRFKTIDECRRT*---
Elapid_Venom_KSPI_04    59 GCGGNANRFKTIDECRRT*---
Elapid_Venom_KSPI_05    59 GCGGNANRFKTIDECRRT*---
Elapid_Venom_KSPI_06    59 GCGGNANRFKTIDECRRT*---
Elapid_Venom_KSPI_07    59 GCGGNANRFKTIDECRRTCAG*
Elapid_Venom_KSPI_08    59 GCGGNANRFKTIDECRRTCAG*
Elapid_Venom_KSPI_09    59 GCGGNANRFKTIDECRRTCAG*
Elapid_Venom_KSPI_10    59 GCGGSANRFKTIDECRRTCAG*
Elapid_Venom_KSPI_11    59 GCGGNANRFKTIGECRRTCAG*
Elapid_Venom_KSPI_12    59 GCGGNANRFKTIDECRRTCAG*
Elapid_Venom_KSPI_13    59 GCGGNANRFKTIDECRRTCAG*
Elapid_Venom_KSPI_14    59 GCGGNANRFKTIDECRRTCAG*
Elapid_Venom_KSPI_15    59 GCGGNANRFKTIDECRRACAG*
Elapid_Venom_KSPI_16    42 GCGGNANRFKTIDECHRACVR*
```

(C) C-type lectins

```
Elapid_Venom_CTL_01    1 MRRFLFVTLGLLVVAFSLNGANGCC-CPRDWLPKNGFCYKVFNDRKNWNDAETFCRKHKP
Elapid_Venom_CTL_02    1 MRRFLFVTLGLLVVAFSLNGANGCC-CPRDWLPKNGFCYKVFNDRKNWNDAETFCRKHKP
Elapid_Venom_CTL_03    1 MRRFLFVTLGLLVVAFSLNGANGCC-CPRDWLPKNGFCYKVFNDRKNWNDAETFCRKHKP
Elapid_Venom_CTL_04    1 MRRFLFVTLGLLVVAFSLNGANGCC-CPRDWLPKNGFCYKVFNDRKNWNDAETFCRKHKP
Elapid_Venom_CTL_05    1 MRRFLFVTLGLLVVAFSLNGANGCC-CPRDWLPKNGFCYKVFNDRKNWNDAETFCRKHKP
Elapid_Venom_CTL_06    1 MGRFLFVTLGLLVVAFSLNGIGANLDCPSSWVSYNVSCYKLFKRMMTWNQAQTYCAEQQE
Elapid_Venom_CTL_07    1 MGRFLFVTLGLLVVAFSLNGIGANLDCPSSWVSYNVSCYKLFKRMMTWNQAQTYCAEQQE
Elapid_Venom_CTL_08    1 MGRFLFVTLGLLVVAFSLNGIGANLDCPSSWVSHNVSCYKLFKRMMTWNQAQTYCAEQQE
Elapid_Venom_CTL_09    1 MGRFLFVTLGLLVVAFSLNGIGANLDCPSSWVSHNVSCYKLFKRMMTWNQAQTYCAEQQE
Elapid_Venom_CTL_10    1 MGRFLLVSLGLLVVAFSLNGIGADHHCPSDWHSFDKFCYKLIKEWKSWSDAEESCIQRQN
Elapid_Venom_CTL_11    1 MGRFLLVSLGLLVVAFSLNGIGADHHCPSDWHSFDKFCYKLIKEWKSWSDAEESCIQRQN

Elapid_Venom_CTL_01   60 GCHLASIHGSEESADLAEYVSDYLSNGGNVWIGLNDPQKQRNWQWTDRSTNSYLTWKQGE
Elapid_Venom_CTL_02   60 GCHLASIHGSEESADLAEYASDYLSNGGNVWIGLNDPQKQRNWQWTDRSTNSYLTWKQGE
Elapid_Venom_CTL_03   60 GCHLASIHGSEESADLAEYASDYLSNGGNVWIGLNDPQKQRNWQWTDRSTNSYLTWKQGE
Elapid_Venom_CTL_04   60 GCHLASIHGSEESADLAEYASDYLSNGGNVWIGLNDPQKQRNWQWTDRSTNSYLTWKQGE
Elapid_Venom_CTL_05   60 GCHLASIHGSEESADLAEYASDYLSNGGNVWIGLNDPQKQRNWQWTDRSTNSYLTWKQGE
Elapid_Venom_CTL_06   61 NCQLASINEVGESVKSSDELSQRLEVL-DVWMGLS--KTKGNWKWSDGSNFTYTSWDEGE
Elapid_Venom_CTL_07   61 NCQLASINEVGESVKLSDELSQRLEVL-DVWMGLS--KTKGNWKWSDGSNFTYTSWDEGE
Elapid_Venom_CTL_08   61 NCQLASINEVGESVKLSDELSQRLEVL-DVWMGLS--KTKGNWKWSDGSNFTYTSWDEGE
Elapid_Venom_CTL_09   61 NCQLASINEVGESVKSSDELSQRLEVL-DVWMGLS--KTKGNWKWSDGSNFTYTSWDEGE
Elapid_Venom_CTL_10   61 SSHLVSIQNWEESFNVKTVISRNLILFTNVWIGLNDPEKTRTWRWSDDSKFDYTSWELRE
Elapid_Venom_CTL_11   61 SSHLVSIQNWEESFNVKTVISRNLILFANVWIGLNDPEKTRTWRWSDDSKFDYTSWELRE

Elapid_Venom_CTL_01  120 PNNLRNNENCVELLSPSGYKNWNDENCASTRAYLCKCRF*-------
Elapid_Venom_CTL_02  120 PNNLRNNENCVELLSPSGYKNWNDENCASTRAYLCKRRF*-------
Elapid_Venom_CTL_03  120 PNNLRNNENCVELLSPSGYKNWNNENCASTRAYLCKCRF*-------
Elapid_Venom_CTL_04  120 PNNLRNNENCVELLSPSGYKNWNDENCASTRAYLCKCRF*-------
Elapid_Venom_CTL_05  120 PNNLRNNENCVELLSPSGYENWNDENCASTRAYLCKCRF*-------
Elapid_Venom_CTL_06  118 PDNFLNNEFCAALSAQSRYLRWNDQNCMFLQPFVCKFKPRSQPENE*
Elapid_Venom_CTL_07  118 PDNFLNNEFCAALSAQSRYLRWNDQNCMFLQPFVCKFKPRSQPENE*
Elapid_Venom_CTL_08  118 PDNFLNNEFCAALSAQSRYLRWNDQNCMFLQPFVCKFKPRSQPENE*
Elapid_Venom_CTL_09  118 PDNFLNNEFCAALSAQSRYLRWNDQNCMFLQPFVCKFKPRSQPENE*
Elapid_Venom_CTL_10  121 PSNADDGKYCVQLSSSSRYLRWKTAKCESKNFFICKM*---------
Elapid_Venom_CTL_11  121 PGNADDGKYCVQLSSSSRYLRWKTAKCESKNFFICKM*---------
```

(D) Snake venom metalloproteinases

```
Elapid_Venom_SVMP_01    1 LERNKGLFSEDYTETHYAPDGREITTSPPVQDHCYYHGYIQNEDESSAVISACDGLKGHF
Elapid_Venom_SVMP_02    1 LERNKGLFSEDYTETHYAPDGREITTSPPVQDHCYYHGYIQNEDESSAVISACDGLKGHF
Elapid_Venom_SVMP_03    1 LERNKGLFSEDYTETHYAPDGREITTSPPVQDHCYYHGYIQNEDESSAVISACDGLKGHF
Elapid_Venom_SVMP_04    1 ??????????????????????????????????????YIQNEDESSAVISACDGLKGHF
Elapid_Venom_SVMP_05    1 ????????????????????????????????????????????????????????????

Elapid_Venom_SVMP_01   61 KHQGETYFIEPLKLSESEAHAIYKDENVEKEDETPQICGVAQTTWESDESIEKTSQLTNT
Elapid_Venom_SVMP_02   61 KHQGGTYFIEPLKLSESEAHAIYKDENVEKEDETPQICGVAQTTWESDESIEKTSQLTNT
Elapid_Venom_SVMP_03   61 KHQGETYFIEPLKLSESEAHAIYKDENVEKEDETPQICGVAQTTWESDESIEKTSQLTNT
Elapid_Venom_SVMP_04   23 KHQGETYFIEPLKLSESEAHAIYKDENVEKEDETPQICGVAQTTWESDESIEKTSQLTNT
Elapid_Venom_SVMP_05    1 ????????????????????????????????????????????????????????????

Elapid_Venom_SVMP_01  121 PEQNRYLQVKKYIEIYMVVDYKMYMNYKRDKSAIKKKVYEMVNTMYTWYIRINNYIVLIG
Elapid_Venom_SVMP_02  121 PEQNRYLQVKKYIEIYMVVDYKMYMNYKRDKSAIKKKVYEMVNTMYTWYIRINNYIVLIG
Elapid_Venom_SVMP_03  121 PEQNRYLQAKKYIEIYMVVDYKMYMNYKRDKSAIKKKVYEMANTMYTWYVRINNYIVLIG
Elapid_Venom_SVMP_04   83 PEQNRYLQVKKYIEIYMVVDYKMYMNYKRDKSAIKKKVYEMVNTMYTWYIRINNYIVLIG
Elapid_Venom_SVMP_05    1 ??????????????????????????????????KKKVYEMANTMYTWYIRINNYIVLIG

Elapid_Venom_SVMP_01  181 LEIWSNGDQIIMHPAANITLDLFGEWRENVLLPRKWNDNTQILTGINLLGNTVGYGYVGT
Elapid_Venom_SVMP_02  181 LEIWSNGDQIIMHPAANITLDLFGEWRENVLLPRKWNDNTQILTGINLLGNTVGYGYVGT
Elapid_Venom_SVMP_03  181 LEIWSNGDQIIMHPAANITLDLFGEWRENVLLPRKWNDNTQILTGINLLGNTVGYGYVGT
Elapid_Venom_SVMP_04  143 LEIWSNGDQIIMHPAANTTLDLFGEWRENVLLPRKWNDNTQILTGINLLGNTVGYGYVGT
Elapid_Venom_SVMP_05   27 LEIWSNGDQIIMHPAANITLDLFGEWRENVLLPRKWNDNTQILTGINLLGNTVGYGYVGT

Elapid_Venom_SVMP_01  241 LCGLATSAAVIQDFSKRTSMVATIMAHELGHNMGIHHDNDTCNCGANICIMSATIHDDPA
Elapid_Venom_SVMP_02  241 LCGLATSAAVIQDFSKRTSMVATIMAHELGHNMGIHHDNDTCNCGANICIMSATIHDDPA
Elapid_Venom_SVMP_03  241 LCGLATSAAVIQDFSKRTSMVATIMAHELGHNMGIHHDNDTCNCGANICIMSATIHDDPA
Elapid_Venom_SVMP_04  203 LCGLATSAAVIQDFSKRTSMVATIMAHELGHNMGIHHDNDTCNCGANICIMSATIHDDPA
Elapid_Venom_SVMP_05   87 LCGLATSAAVIQDFSKRTSMVATIMAHELGHNMGIHHDNDTCNCGANICIMSATIHDDPA

Elapid_Venom_SVMP_01  301 FQFSSCSVQQYQEFLLRERPQCILNKPLSTDIVSPPVCGNYLVEVGEECDCGSPEDCQSA
Elapid_Venom_SVMP_02  301 FQFSSCSVQQYQEFLLRERPQCILNKPLSTDIVSPPVCGNYLVEVGEECDCGSPEDCQSA
Elapid_Venom_SVMP_03  301 FQFSSCSVQQYQEFLLRERPQCILNKPLSTDIVSPPVCGNYLVEVGEECDCGSPEDCQSA
Elapid_Venom_SVMP_04  263 FQFSSCSVQQYQEFLLRERPQCILNKPLSTDIVSPPVCGNYLVEVGEECDCGSPEDCQSA
Elapid_Venom_SVMP_05  147 FQFSSCSVQQYQEFLLRERPQCILNKPLSTDIVSPPVCGNYLVEVGEECDCGSPEDCQSA

Elapid_Venom_SVMP_01  361 CCDAATCKLQQEAQCDSEECCEKCKFKKAGAECREAKDDCDLPELCTGQSAECPTDRFQR
Elapid_Venom_SVMP_02  361 CCDAATCKLQQEAQCDSEECCEKCKFKKAGAECREAKDDCDLPELCTGQSAECPTDRFQR
Elapid_Venom_SVMP_03  361 CCDAATCKLQQEAQCDSEECCEKCKFKKAGAECREAKDDCDLPELCTGQSAECPTDRFQR
Elapid_Venom_SVMP_04  323 CCDAATCKLQQEAQCDSEECCEKCKFKKAGAECREAKDDCDLPELCTGQSAECPTDRFQR
Elapid_Venom_SVMP_05  207 CCDAATCKLQQEAQCDSEECCEKCKFKKAGAECREAKDDCDLPELCTGQSAECPTDRFQR

Elapid_Venom_SVMP_01  421 NGHPCQNNQ
Elapid_Venom_SVMP_02  421 NGHPCQNNQ
Elapid_Venom_SVMP_03  421 NGHPCQNNQ
Elapid_Venom_SVMP_04  383 NGHPCQNNQ
Elapid_Venom_SVMP_05  267 NGHPCQNNQ
```
